# Activity-based costing for HIV, primary care and nutrition services in low- and middle-income countries: A systematic literature review and synthesis

**DOI:** 10.52872/001c.29068

**Published:** 2021-10-25

**Authors:** Diana Bowser, Anna Sombrio, Neto Coulibaly, Noah Mark

**Affiliations:** 1 The Heller School for Social Policy and Management, Brandeis University

**Keywords:** systematic review, low- and middle-income countries, economics, activity-based costing, primary care, nutrition, HIV

## Abstract

**Background:**

This study is a systematic literature review of HIV, nutrition, and primary care activity-based costing (ABC) studies conducted in low- and middle-income countries. ABC studies are critical for understanding the quantities and unit costs of the activities and resources for specific cost functions. The results of ABC studies enable governments, funders, and policymakers to utilize costing results to make efficient, cost-effective decisions on how to allocate scarce resources.

**Methods:**

We followed the Preferred Reporting Items for Systematic Reviews and Meta-Analyses (PRISMA) methodology for systematic literature reviews. Key search terms included: (1) activity-based costing and time-driven activity-based costing, (2) cost of services, (3) HIV interventions OR (4) primary health care. Terms were searched within article titles and abstracts in PubMed, EconLit, and Scopus.

**Results:**

1,884 abstracts were screened and reduced to 57 articles using exclusion criteria. After a full text review, 16 articles were included in the final data synthesis. Findings were used to classify costs into relevant and common inputs for activity-based costing. All costs were converted to unit cost (cost per patient) and inflated to January 2020 USD. The largest unit cost across nutrition services was training (US$194.16 per patient, 34.6% of total unit cost). The largest unit cost for HIV was antiretroviral therapy (ART) (US$125.41, 71.0%). The largest unit cost for primary care services was human resources (US$84.78, 62.5%). Overall costs per patient for HIV services were US$176.71, US$135.67 for primary care services, and US$561.68 for nutrition services. The costing results presented suggest that spending on HIV exceeds the actual cost of HIV services.

**Conclusions:**

This is the first systematic literature review to summarize the costs of HIV, primary care, and nutrition services across activity-based costing studies. While there was a wide variation in the study designs and economic methods, many of the input cost categories were similar. With the increasing number of costing studies in countries around the world, understanding trends in costs by function and service can lead to greater efficiency in the implementation of HIV, primary care, and nutrition programs.

## INTRODUCTION

Despite the abundance of costing studies and cost analyses for various types of health care programs in low- and middle-income countries (LMICs), there remains a gap in results from two types of costing techniques: activity-based costing (ABC) and time-driven activity-based costing (TD-ABC). ABC refers to costing studies that identify costs needed to perform certain activities to understand the “quantities and the unit costs of the activities and the resources deployed for the individual cost objects.”^1^ TD-ABC refers to a similar costing technique, but with the perspective of accounting for time and resource use at the level of the individual patient.^2^

While the literature provides a plethora of costing studies on many types of health care programs, including HIV/ AIDS programs and primary health care, costing studies that specifically state that they utilize ABC or TD-ABC methods are found less frequently. The Global Health Costing Consortium^3^ summarises many types of costing studies on HIV from various perspectives, but does not provide the types of costing method used to collect the data (top-down, bottom-up, ABC, TD-ABC). In addition, there are many types of costing studies, each with its own methods and mechanisms of calculating costs, making cross-country or cross-study comparisons difficult.^4–7^ Understanding costing data for essential services such as HIV and other primary health care services from the perspective of specific cost categories and the individual perspective is immensely important as countries continue to strive to achieve Universal Health Coverage, even during the COVID-19 pandemic.^8^

Information about the cost per HIV, primary care, and nutrition service, as well as the cost inputs for each of these services, is critical to donors and governments who play pivotal roles in funding future programs.^9^ These three services are the focus of this systematic literature review because of the importance of finding efficiencies in the provision of HIV services^10,11^ and the integration of primary care, nutrition and HIV.^12,13^ Our objective was to critically assess the current evidence from the literature on the ABC and TD-ABC costing results for HIV, primary care, and nutrition services in LMICs to assist governments and donors in making key funding decisions. While there are a plethora of costing techniques, ABC and TD-ABC show promise as more reliable forms of costing for these types of services, providing important information to enable government and other policymakers to make efficient, cost-effective decisions on how to allocate limited resources.^2,14^

## METHODS

We conducted a systematic literature review (SLR) to investigate the results from studies on ABC and TD-ABC for HIV services and primary care in LMICs. The review followed the Preferred Reporting Items for Systematic Reviews and Meta-Analyses (PRISMA) guidelines.^15^ On April 11, 2020, we submitted the systematic review protocol to the Prospective Register of Systematic Reviews (PROSPERO) prior to the start of this review (ID Number: CRD42020179576). Scopus was used instead of Web of Science as Scopus is generally considered an appropriate alternative to Web of Science for systematic reviews.

### IDENTIFICATION

We searched key databases (PubMed, EconLit, and Scopus) using search terms related to (1) TD-ABC studies (e.g. activity-based cost OR bottom-up cost OR unit costing), (2) cost of services, and (3) HIV interventions OR (4) primary health care.

We developed search strategies with a professional librarian to ensure accuracy and validity. We first developed a search strategy for PubMed using a combination of medical subject headings (MeSH) terms and keywords for each concept, incorporating adapted search filters for TD-ABC and other related costing methodologies, cost of services, HIV interventions, and primary health care. Next, we adapted the PubMed search strategy to the other databases using their search terms. The complete search strategy is shown in Table 1. It is worth noting that while we initially included time-driven activity-based costing as a key search term, only a few of these costing studies were identified through the search. In addition, while nutrition was not included as a search term, as noted below, several primary care studies focused on nutrition interventions were included in the results.

### SCREENING

We then applied the study selection of inclusion and exclusion criteria to the list of studies identified. We included all studies reporting HIV or primary health care costs, including nutrition, in LMICs using TD-ABC or a related costing methodology. While TD-ABC was initially the focus of the SLR, this costing methodology has not been conducted frequently in LMICs. The complete inclusion and exclusion criteria are shown below. We restricted our analysis to those studies between January 01, 2000, and March 20, 2020.

Inclusion:

1. Study of primary health care or HIV interventions AND (A) AND (B) with (C or D)
  - TD-ABC studies (e.g. activity-based cost OR bottom-up cost OR unit costing)
  - Cost of services
  - Process maps
  - Management decisions
2. Data from observational studies, population studies, claims analyses, or case series
3. Published between January 01, 2000 and March 20, 2020
4. English, French, or Spanish language
5. Recognized scientific literature (published books, peer-reviewed articles or chapters in books, indexed proceedings, government reports, reports of international organizations, dissertations, or RSV protocols)
6. Low- and middle-income countries

Exclusion:

1. Studies focused on interventions that are not related to primary health care or HIV interventions
2. Studies not in low- and middle-income countries or where data for those locations cannot be separated out.
3. Published before 2000
4. Editorials, newspapers, and other pieces of popular media
5. Book reviews or organizational reports with no original data
6. Other systematic reviews with no original data
7. References that are missing abstracts

Following PRISMA guidelines, two authors (AS and NC) evaluated all potential articles independently during both the title and abstract screening and full text eligibility phases. The senior investigator (DB) resolved conflicts.

### ELIGIBILITY

We included all studies that contain HIV, primary healthcare, and nutrition costs in LMICs since 2000. We developed a data extraction table that allowed for similar cost categories to be extracted from each included study. In addition, we extracted the following characteristics from all included studies: authors, year published, journal, costing methodology, dates of study data, cost categories and specific resources, and cost analysis results. We then categorized cost data into categories based on cost inputs/ingredients and service costs. Where possible, we compared similar cost categories across studies and unit utilizing cost per visit and per patient. All authors conferred together about inconsistencies and adjustments to the data extraction.

### ANALYSIS OF INCLUDED ARTICLES

We inflated all reported costs from the study’s costing year to January 2020 USD using the Consumer Price Index for All Urban Consumers for Medical Care.^16^ For studies that did not report a costing year, we used the midpoint of the study collection dates. All cost data that were not already in USD were converted to USD using the exchange rate for the date the cost data were presented in the study.

The sample size in the included studies refers to the patients who were treated for the services provided. We extrapolated the sample size (number of patients served) for four of the six clinics in the study done by Rout et al.^17^ by determining the average number of visits per patient from the data provided for the Ahmednagar and Jalna clinics. For studies that did not report per patient unit costs per cost category, we calculated the per patient unit cost by dividing the total costs for each cost category, and the total number of patients served.

With-in study and cross-study weighting of final per capita unit costs were calculated to account for variation in size of sites reported within each study and total study sample sizes. Within-study weighted averages for per patient unit cost were calculated for studies that had multiple sites that reported cost data. The weights were based on the sample sizes (number of patients served) for study sites. Additionally, we calculated cross-study weights to determine the average unit cost per patient across each of the cost categories (human resources, equipment and capital, laboratory, supplies, antiretroviral therapy (ART) and medicines, training, logistics, and nutrition service delivery). Both within-study and cross-study weights were calculated using the number of patients per site and total patients in the study, respectively. For studies in which nutrition or HIV services were funded from multiple sources, we consolidated cost data to get total expenditure per cost category. We ultimately calculated the cross-study total unit cost per patient by adding the cross-study unit cost per patient across all relevant cost categories. Definitions of cost categories are standard definitions for categories related to health care service delivery. The category for ART and medicines refers to the cost of the medicines and treatment and not the personnel associated with distributing the medicines (as these costs were captured in other cost categories). All data were compiled and analyzed using Microsoft Excel Version 1808 (Redmond, WA).

## RESULTS

### STUDY SELECTION

**Figure 1.**
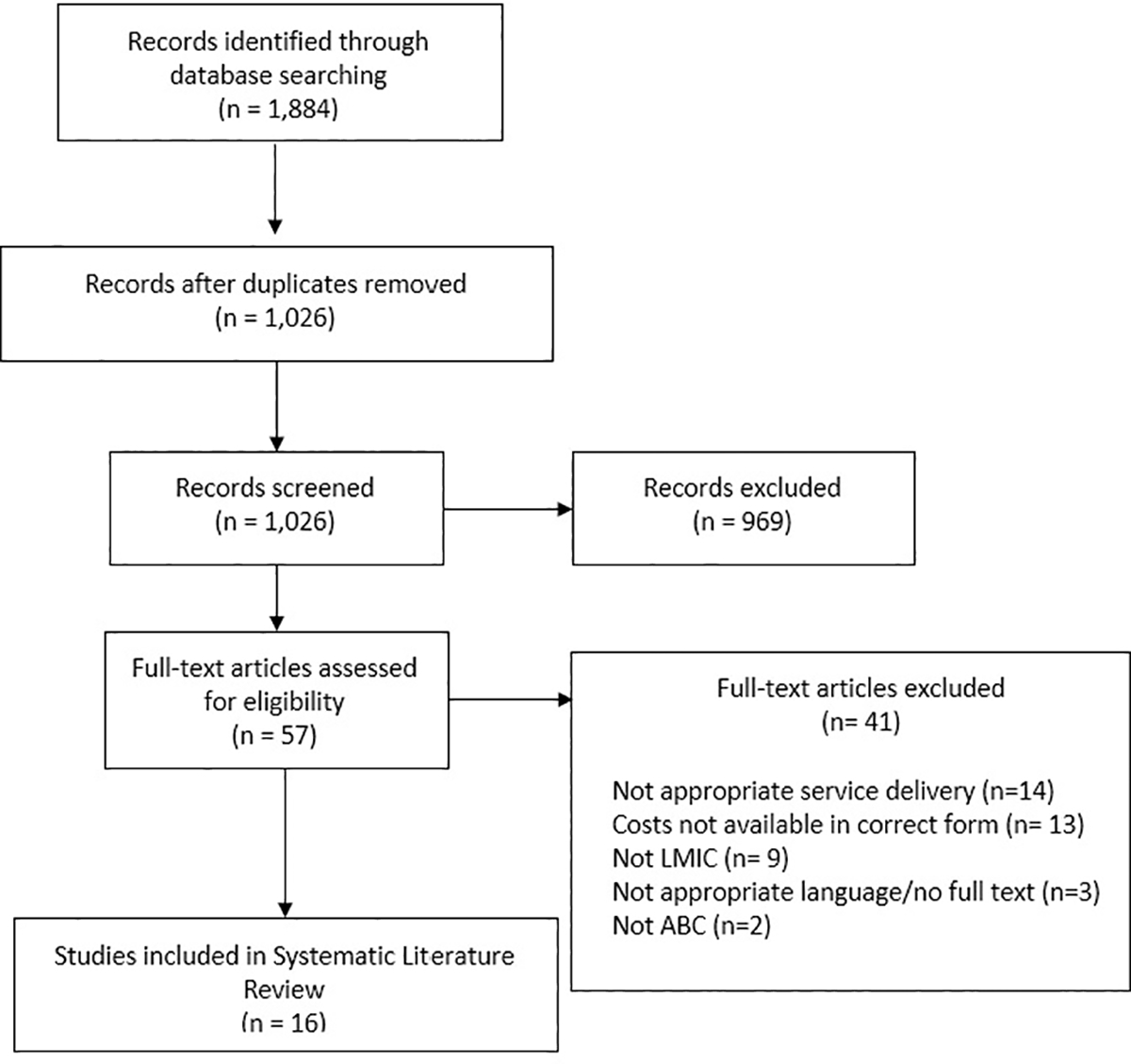
PRISMA Diagram

Our initial literature search returned 1,884 citations. After eliminating duplicates, 1,026 unique references remained for the title and abstract screening. The initial screening found that 969 unduplicated articles did not meet inclusion criteria. We then screened the remaining 57 articles at the full text review stage and excluded an additional 41 articles, mainly for not presenting the costing results in a form that could be used in the synthesis or not presenting service cost for HIV, primary care, or nutrition. The PRISMA diagram (Figure 1) reports the results of each phase of the review. In the end, 16 articles met the eligibility criteria for inclusion in this review. There were no additional studies identified from other methods.

### STUDY CHARACTERISTICS

Table 2 reflects the study characteristics of this review. The studies included were conducted in LMICs, classified by the World Bank: 42% were from low income and 58% from low-middle-income countries. All included studies reported costs from the following health focus areas: 5 from HIV (42%) and seven from nutrition (58%). Nearly all studies 10/12 (83%) used activity-based costing methods, and 2/12 (17%) used bottom-up costing methods. While this systematic literature review included TD-ABC costing method, as shown in Table 2, no TD-ABC studies remained upon final eligibility analysis, as most of those studies were eliminated because they reported hospital-level costs or were conducted in high-income countries.

### COST SUMMARY AND ANALYSIS

Table 3 shows the variation in unit costs per year (per patient) across five main ABC inputs: human resources, equipment and capital, laboratory, supplies, and ART and medicines. Unit costs for HIV-provision activities across studies vary both within and across the stated cost input categories. The highest per patient cost was reported from an RCT Kampala study in Uganda, with human resource per patient cost of US$782.51.^18^ The lowest per patient costs was reported for two clinics in Zambia, reporting human resource per patient costs of US$3.11.^19^ The cross-study weighted human resource per patient cost for HIV services is US$26.60. Equipment and capital costs are the lowest cost category with a cross-study average of US$1.68, ranging from a low of US$0.16 in one clinic in Zambia (Clinic 10) to a high of US$25.08 in one state in India (Jalna State).^17,19^ The cross-study average for laboratory cost is US$19.01, ranging from a low of US$1.08 in Malawi to a high of US$185.56 in Burkina Faso.^14,20^ The cross-study average for supply costs are US$4.01, ranging from a low of US$0.59 in Zambia to a high of US$26.41 in India.^17,19^ The cross-study average for ART and medicines cost is US$125.41 per person, with a high of US$547.63 for India, Pandharpur, and a low of US$1.89 for Malawi, Neno District.^14,17^ The total per patient unit cost of implementing HIV services that includes human resources, equipment and capital, laboratory, supplies, and ARTs and medicines is US$176.71 per patient per year.

Only one HIV study reported technology and training costs, with the calculated per patient unit costs of US$2.49 for technology and US$5.27 for training.^14^ Technology accounted for 32% of the costs across these two categories, and training accounted for the remaining 68%. Overall, these two cost categories accounted for a total average per patient unit cost of US$7.76. Only three studies reported travel costs – one HIV study and two nutrition studies. The calculated per patient unit cost for the HIV study is US$8.03.^14^ The per patient unit costs for the Kenya nutrition study are US$10.75 and US$9.71 for NGO 1 and NGO 2, respectively.^21^ The per patient unit costs for the Mali nutrition study are US$18.29 and US$51.10 for intervention and control areas, respectively.^22^ The cross-study average for transportation in the two nutrition studies is US$13.55 per person.

**Figure 2.**
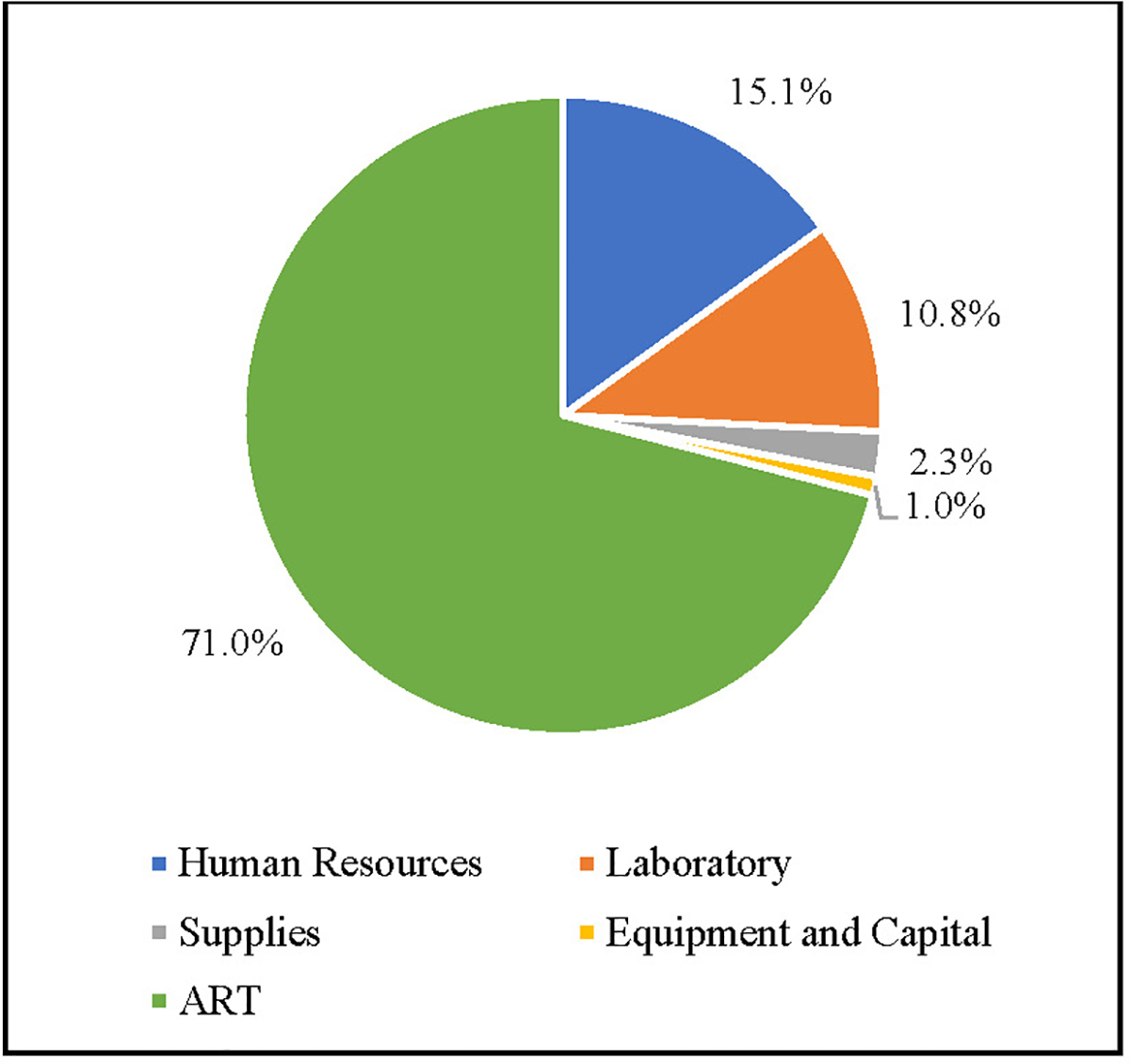
Cross-Study Average Per Patient Unit Cost Distribution for HIV Services (%) Note: ART denotes antiretroviral therapy

As shown in Figure 2, using data from the HIV studies included in this SLR, the ART cross-study average unit cost category accounts for the largest share of per patient unit costs for HIV services (71.0%), followed by human resources (15.1%), laboratory (10.8%), supplies (2.3%), and equipment and capital (1.0%).

We ultimately identified seven nutrition-related studies that matched the inclusion criteria for our literature review. Table 4 shows the variation in unit costs per year (per patient) across six main activity-based costing inputs: human resources, equipment and capital, training, logistics, service delivery, and supplies. Unit costs for nutrition provision activities across studies vary both within and across the stated cost input categories. The highest human resources per patient cost is for the cost reported from Zimbabwe, LIG site, with human resource per patient cost reported as US$2,166.71.^23^ The lowest per patient cost is reported for Kenya, NGO 1, with a per patient unit cost of US$4.00.^21^ The cross-study human resource per patient unit cost for nutrition services was US$120.17. The equipment and capital cost category is the lowest-cost with an average cross-study per person unit cost of US$1.13. The highest reported unit cost in this category is for Bangladesh, Inpatient Treatment, with a unit cost of US$1.46.^24^ The lowest unit cost is reported for Kenya, NGO 2, with a cost of US$0.72.^21^ The training cost category is the highest cost by a wide margin, with an average per person unit cost of US$194.16. The highest unit cost within this category is reported by Zimbabwe, LIG, at a per person unit cost of US$1,152.26.^23^ The lowest reported cost is for Mali, Intervention, with a reported unit cost per person of US$16.88. The average cross-study per person unit cost within the logistics cost category is US$27.19. The highest unit cost reported for logistics is for Mali, Control, with a per person cost of US$44.39.^25^ The lowest unit cost reported is for Peru, Intervention Area, with a per person cost of US$4.75.^26^ In the service delivery cost category, the highest reported cost is for Peru, Control Area, with a unit cost of US$304.16 per person. The lowest cost is reported for Chad, Food Assistance, with a unit cost of US$43.74 per person. The average unit cost for service delivery is US$69.60 per person. In the supplies cost category, unit costs show a large range, with a high of US$830.88 per capita reported for Chad, Food Assistance, and a low of US$2.53 per capita reported for Kenya, NGO 1.^21,27^ Overall, the total per patient unit cost of implementing nutrition services is US$561.68.

**Figure 3.**
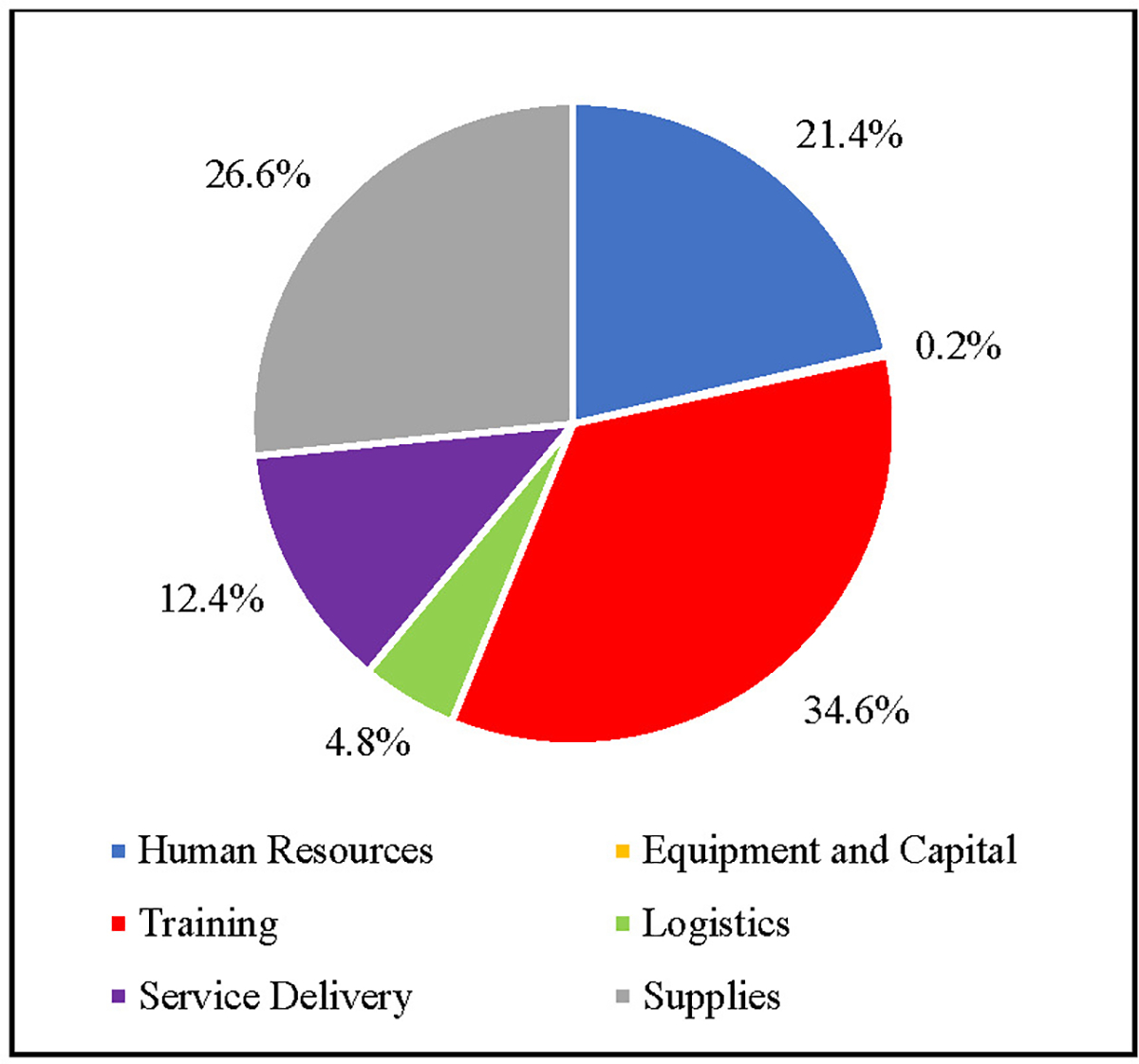
Cross-Study Average Per Patient Unit Cost Distribution for Nutrition Services (%) Note: At 0.2%, the color shading representing Equipment and Capital is undetectable in this figure

Figure 3 shows the portion of mean unit cost per capita attributed to each cost category for nutrition services. Unlike the HIV studies, the nutrition studies have the largest percentage of total unit costs per patient attributed to training at 34.6%. Supplies account for the second largest portion of total unit costs at 26.6%. Human resources account for the third largest portion of total unit costs at 21.4%. Service delivery account for the fourth largest portion of total unit costs per person at 12.4%. The logistics category account for 4.8% of the total unit cost per capita. Equipment and capital account for the smallest portion of total mean unit cost per capita at 0.2%.

We ultimately identified four primary care-related studies that matched the inclusion criteria for our literature review. Table 5 shows the variation in unit costs per year (per patient) across six main activity-based costing inputs: human resources, equipment and capital, supplies, consumables/medicines, laboratory, and miscellaneous. Unit costs for primary care provision activities across studies vary both within and across the stated cost input categories. The highest human resources per patient cost is for the cost reported from India, Primary Health Centers with a per patient cost reported as US$153.14.^28^ The lowest per patient cost is reported for Burkina Faso, Design Phase at US$0.07.^29^ Under the equipment and capital cost input, the highest cost is reported for India, Primary Health Centers at a per person unit cost of US$27.23.^28^ The lowest cost is reported for Pakistan, Gov’t PHC; PNA and Severe PNA at a per person unit cost of US$0.10.^30^ Under the supplies input category, the highest cost is reported for India, Primary Health Centers at a per person unit cost of US$14.10.^28^ The lowest per person unit cost is reported for Pakistan, AKHSP PHC; PNA and Severe PNA at a cost of US$0.01.^30^ Under the consumables/medicines cost category, the highest cost is reported for India, Primary Health Centers at a per person unit cost of US$55.02.^28^ The lowest cost is reported for Burkina Faso, Design Phase at a per person unit cost of US$0.003.^29^ In the laboratory cost category, the highest per person unit cost is reported for India, Primary Health Centers at a cost of US$7.90.^28^ The lowest per person unit cost is reported for India, Patna and Mumbai at a cost of US$0.04.^31^ In the miscellaneous cost category, the highest per person unit cost is reported for India, Mehsana with a cost of US$19.71.^31^ The lowest per person unit cost is reported for Gov’t PHC; PNA, Severe PNA, and AKHSP PHC; PNA at a cost of US$0.004.^30^ Overall, the total per patient unit cost of implementing primary care services is US$135.67.

Common miscellaneous costs in Patna, Mumbai, and Mehsana sites included call center costs and telecom costs. Specific to Patna are other operational costs not specified. Mumbai and Mehsana shared a common cost category of SMS costs. Mumbai is the only site to also incur information, education and communication (IEC) activity costs. In India for Primary Health Centers and Community Health Centers, IEC material is included as an additional cost category. Lastly, miscellaneous costs are reported for all the Pakistan sites but are not specified further than that.

Only the Deo et al. India study^31^ report training costs with an average per person unit cost of US$1.20. Per person unit costs for the Patna, Mumbai, and Mehsana sites are US$0.05, US$2.69, and US$1.01, respectively. This is also the only study to report information technology (IT) costs, with the per person unit cost for Mehsana reported as US$0.85. The Burkina Faso study^29^ is the only study to report costs for transportation, with an average per person unit cost of US$0.30. The per person unit costs for the Design Phase and Implementation Phase are US$0.19 and US$0.41, respectively.

**Figure 4.**
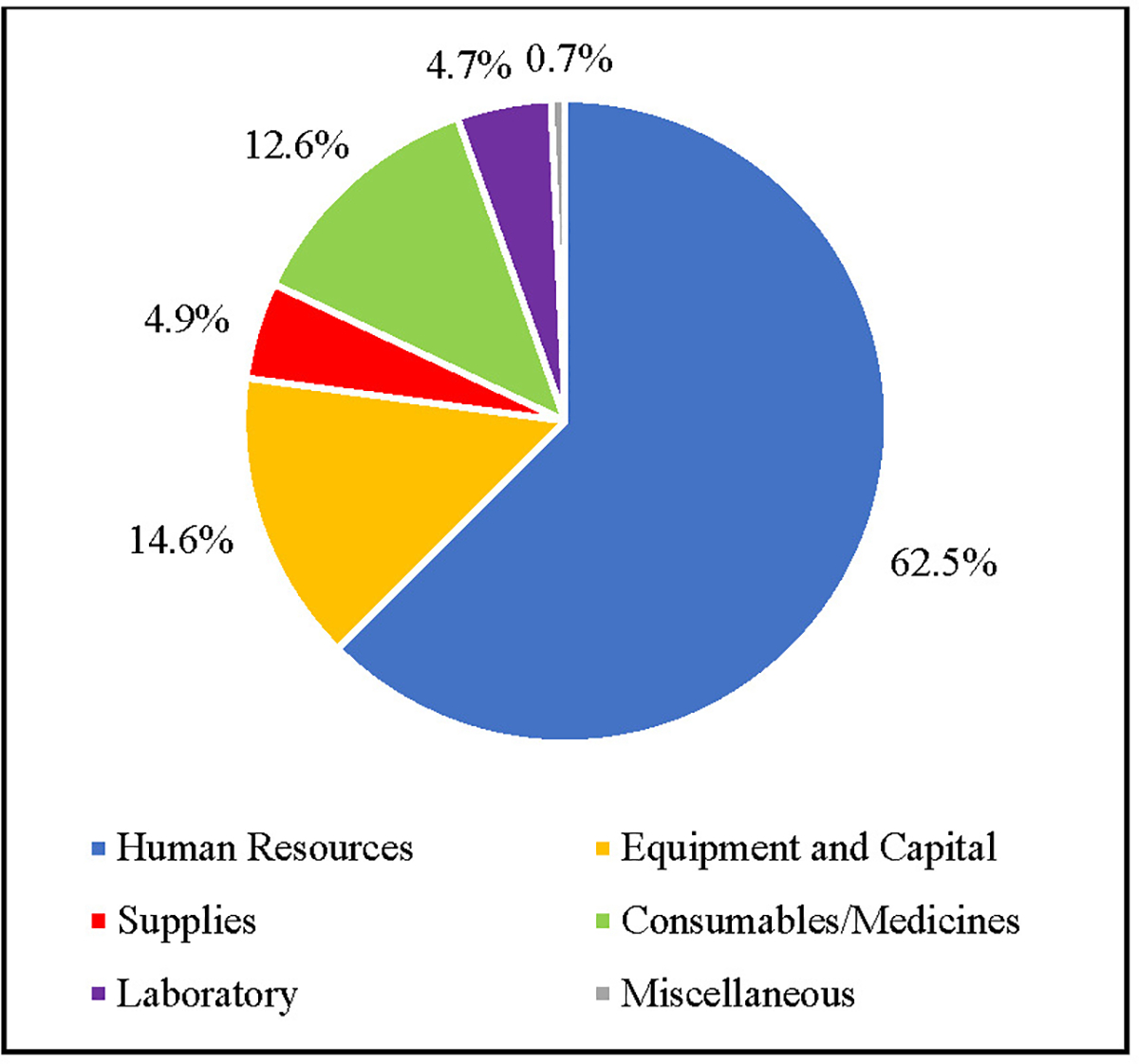
Cross-Study Average Per Patient Unit Cost Distribution for Primary Care Services (%)

Figure 4 shows the portion of mean unit cost per capita attributed to each cost category for primary care services. Similar to the HIV studies, the primary care studies have the largest percentage of total unit costs per patient attributed to human resources at 62.5%. Equipment and capital accounted for the second largest portion of unit cost per patient at 14.6%. Following that is consumables/medicines at 12.6%, supplies at 4.9%, laboratory at 4.7%, and finally miscellaneous at 0.7% of total per patient unit costs.

Table 6 shows the variation in unit costs per year across the overhead cost category for HIV, nutrition, and primary care studies. Unit costs vary widely across countries in HIV studies, with an average unit cost for the overhead of US$37.20 per person. The highest unit cost per person is reported for Burkina Faso, Yerelon Clinic, with a cost of US$370.13 per person. The lowest unit cost is reported for Zambia, Clinic 1, with a cost of US$0.20 per person. Unit costs for overhead have a lower range across nutrition studies, with an average unit cost for the overhead of US$9.00 per person. The highest unit cost for overhead is reported for Pakistan, Control, with a cost per person of US$39.56. The lowest cost is reported for Kenya, NGO 1, with a cost of US$0.48 per person. Overhead unit costs across primary care studies are relatively low – the highest cost per person for overhead is reported for India, Primary Health Centers at the cost of US$14.10. The lowest cost per person is reported for AKHSP PHC PNA and Severe PNA at the cost of US$0.01.

## DISCUSSION

This is one of the first systematic literature reviews to summarize the activity-based costs for HIV services, primary care services, and nutrition services across LMICs. The results of the synthesis provide useful key per patient costs for HIV services, primary health care services, and nutrition services for policy makers, implementers, and government officials. The results also show that while there was an abundance of costing studies (we identified 1,844 upon our initial search), only a small number of studies reported activity-based costs that could be compared across similar input categories.

While there have been other systematic literature reviews concerning HIV, primary care, and nutrition services, these reviews have focused on specific populations and/or therapies. For example, a study in Asia and Eastern Europe examined spending on HIV across a number of studies, focusing on priority populations.^32^ A study in sub-Saharan Africa examined the cost of ART delivery strategies.^33^ Other studies have summarized the cost-effectiveness of nutrition studies.^34^ This study is novel in that it summarizes the actual cost for different cost categories and services across several studies, providing useful planning and allocation information for program implementers.

Some of the articles included in this systematic literature review are also included in the Global Health Cost Consortium database.^3^ This database is useful, and one is able to sort by intervention type as well as country and cost perspective. The database, however, does not allow one to search by the type of method used in each costing study, which may vary even within the cost perspective. This study complements the studies in the Global Health Cost Consortium by searching systematically for HIV or primary care studies that use TD-ABC or other similar costing methodology. The costing methodology for calculating unit costs within the Global Health Cost Consortium is similar to the methods used in this synthesis.

While donors and funders spend billions of dollars per year on HIV, primary care, and nutrition services in LMICs, they are often unaware of the actual cost of providing these services. In 2018, donors spent US$9.5 billion on HIV services alone.^35^ The results above from this study summarize studies across twelve LMICs, calculating the unit cost per patient of providing HIV, primary care, and nutrition services. These costing results should be useful to donors and policy makers as they compare their spending to the number of patients served. For example, given that the unit cost per patient for HIV services calculated above is US$176.71 and there are 38 million individuals living with HIV (as of 2019),^36^ this would equate to close to US$6.7 billion dollars needed for HIV services (not including overhead). This suggests that spending on HIV exceeds the actual cost of HIV services.

Overhead cost calculations are difficult to implement in many costing studies. While only several studies reported overhead costs, the rates provided above give some evidence of overhead costs for HIV, primary care, and nutrition programs. For costing studies, it is important to calculate overhead costs to understand the additional costs, above service costs, that are related to delivering a service. Since overhead costs are difficult to calculate in health care settings, especially government-funded health services, the summary of overhead costs in this systematic literature review is useful to researchers, policy makers, and leaders.

In addition to summarizing an overall unit cost for HIV, primary care, and nutrition services, the results above also suggest that there is a wide variation within and across countries regarding the costs in the categories highlighted in this study. For human resources alone, the variation is quite significant and there are some interesting trends that could be explored further. The variation in costs across all categories warrants further research, examining some of the factors that impact this variation, including geographic distribution, urban/rural location, donor-supported versus government-supported sites, and/or severity of illness of the patients in the different locations. In particular, the geographic distribution is interesting and needs to be understood in combination with knowledge of and learning around TD-ABC methodology in specific geographic areas.

An initial aim of this systematic literature review was to understand the contribution to the health policy and economic field from not only activity-based costing and bottom-up costing techniques, but also time-driven activity-based costing. The advantage of the time-driven activity-based costing methodology is that the costs are based on the actual time providers and other health care personnel are with the patient. Using the care process as the input reduces the inaccuracy of other costing methodologies, where estimates are based on expenses to certain cost categories (such as supplies, human resources, etc.).^2^ TD-ABC studies have not been conducted frequently in the settings included in this review. Of the studies that were reviewed for inclusion in this analysis, only six studies were initially included in the full text review. Of those six, four were excluded because they were not from a low- or middle-income setting, one because it was inpatient care, and one because access to the full text article was not available. This indicates that more needs to be done to understand how to better implement TD-ABC in LMICs.

## CONCLUSION

Our study provides an important lens on and analysis of recent literature on the economic distribution of providing HIV, primary care, and nutrition services to people in LMICs. Data from this study reflect a wide variation in the costs of providing these services and may give direction to future research studies on interventions in HIV, primary care, and nutrition areas. Our findings will contribute to determining what aspects of care provision are the costliest to help reduce unnecessary spending in certain areas and reallocate dollars to the most important areas. This analysis should help to demonstrate the importance of activity-based costing studies to inform decision makers on implementing programs to help combat HIV, general health issues, and malnutrition in their respective countries.

## Supplementary Material

Supplementary files

## Figures and Tables

**Table 1.** Search Strategies for Key Databases

PubMed	Scopus	EconLit
(“TD-ABC” OR “TDABC” OR “activity-based cost*” OR “bottom-up cost*” OR “unit costing” OR “patient-level cost*”) AND ((“Economics”[MeSH] OR “economics”[subheading] OR Economic* OR Cost* OR Expenditure* OR Accounting) OR (“Process map” OR “Process Assessment, Health Care”[MeSH]) OR (“Equipment and supplies”[MeSH] OR “Health Services Administration”[MeSH] OR “organization and administration”[subheading] OR “Task Performance and Analysis”[MeSH] OR “Workflow”[MeSH] OR “Job Satisfaction”[MeSH] OR Manag* OR Staffing OR Equipment OR Supplies OR Personnel OR schedul* OR “wait time*” OR “prescrib* pattern*” OR satisfaction)) AND (“2000/01/01”[PDat] : “2020/03/20”[PDat])	TITLE-ABS-KEY ((“TD-ABC” OR “activity-based cost*” OR “bottom-up cost*” OR “unit costing” OR “patient-level cost*”) AND (health* OR medic*) AND (economic* OR cost* OR expenditure* OR accounting OR “Process map” OR manag* OR staffing OR equipment OR supplies OR personnel OR schedul* OR “wait time*” OR “prescrib* pattern*” OR satisfaction)) AND (PUBYEAR > 1999) AND (LANGUAGE (English OR French OR Spanish))	“TD-ABC” OR “TDABC” OR “activity-based cost*” OR “bottom-up cost*” OR “unit costing” OR “patient-level cost*”

**Table 2.** Summary of Studies

First Author	Year	Country	Income Level	Costing Method	Health Focus	Study Period

Chou	2007	Uganda	Low-Income	ABC	HIV	2005–2006
Cianci	2014	Burkina Faso	Low-Income	Bottom Up	HIV	2010
Levin	2019	Kenya	Lower-Middle Income	ABC	Nutrition	2011–2013
McBain	2017	Malawi	Low-Income	ABC/CEA	HIV	2013–2014
Puett	2013	Chad	Low-Income	ABC/CEA	Nutrition	2010
Puett	2013	Bangladesh	Lower-Middle Income	ABC/CEA	Nutrition	2010
Puett	2014	Zimbabwe	Lower-Middle Income	ABC/CEA	Nutrition	2008–2011
Rogers	2019	Pakistan	Lower-Middle Income	ABC/CEA	Nutrition	2015–2016
Rogers	2018	Mali	Low-Income	ABC/CEA	Nutrition	2015–2016
Rout	2019	India	Lower-Middle Income	Bottom Up	HIV	2013–2015
Tucker	2020	Zambia	Lower-Middle Income	ABC	HIV	2015–2016
Waters	2006	Peru	Lower-Middle Income	ABC	Nutrition	2000–2001
Deo	2019	India	Lower-Middle Income	ABC	Primary Care	2014–2016
Beauge	2018	Burkina Faso	Low-Income	ABC	Primary Care	2014–2016
Prinja	2016	India	Lower-Middle Income	Bottom Up	Primary Care	2012–2013
Hussain	2006	Pakistan	Lower-Middle Income	ABC	Primary Care	2000–2001

Notes: ABC denotes activity-based costing; CEA denotes cost-effectiveness analysis.

**Table 3.** Annual Per Patient Costs for HIV Studies by Input Cost Categories (In US$, 2020)

First Author (Year)	Country	Type of Cost Unit (Health Center, Department)	Sample Size	Mean Per Patient Unit Cost

**Human Resources**				

Rout (2019)	India	Ahmednagar	58,393	7.62
Rout (2019)	India	Jalna	1,500	137.23
Rout (2019) ^†^	India	Bhandara	13,258	28.21
Rout (2019) ^†^	India	Kolhapur	32,122	13.87
Rout (2019) ^†^	India	Akola	3,774	86.24
Rout (2019) ^†^	India	Pandharpur	5,762	61.21
*Within Study Average*				**18.71**
Chou (2007)	Uganda	Kampala RCT Study	1,420	**782.51**
McBain (2017)	Malawi	Neno District	6,541	**140.10**
Cianci (2014)	Burkina Faso	Direct/Yerelon Clinic	187	69.02
Cianci (2014)	Burkina Faso	Non-Direct/Yerelon Clinic	187	68.27
Within Study Average*				**68.65**
Tucker (2020)	Zambia	Clinic 1	9,104	4.33
Tucker (2020)	Zambia	Clinic 2	8,050	5.44
Tucker (2020)	Zambia	Clinic 3	6,410	4.74
Tucker (2020)	Zambia	Clinic 4	5,127	3.15
Tucker (2020)	Zambia	Clinic 5	4,597	5.57
Tucker (2020)	Zambia	Clinic 6	3,094	4.30
Tucker (2020)	Zambia	Clinic 7	3,080	4.21
Tucker (2020)	Zambia	Clinic 8	1,076	9.23
Tucker (2020)	Zambia	Clinic 9	873	3.11
Tucker (2020)	Zambia	Clinic 10	477	3.11
*Within Study Average*				**4.67**
*Cross-Study Human Resources Average*				**26.60**
**Equipment and Capital**				

Rout (2019)	India	Ahmednagar	58,393	0.47
Rout (2019)	India	Jalna	1,500	25.08
Rout (2019) ^†^	India	Bhandara	13,258	2.40
Rout (2019) ^†^	India	Kolhapur	32,122	0.56
Rout (2019) ^†^	India	Akola	3,774	9.23
Rout (2019) ^†^	India	Pandharpur	5,762	4.47
*Within Study Average*				**1.53**
Chou (2007)	Uganda	Kampala RCT Study	1,420	**15.40**
McBain (2017)	Malawi	Neno District	6,541	**7.60**
Tucker (2020)	Zambia	Clinic 1	9,104	0.61
Tucker (2020)	Zambia	Clinic 2	8,050	0.57
Tucker (2020)	Zambia	Clinic 3	6,410	0.32
Tucker (2020)	Zambia	Clinic 4	5,127	0.29
Tucker (2020)	Zambia	Clinic 5	4,597	0.39
Tucker (2020)	Zambia	Clinic 6	3,094	0.42
Tucker (2020)	Zambia	Clinic 7	3,080	2.67
Tucker (2020)	Zambia	Clinic 8	1,076	3.67
Tucker (2020)	Zambia	Clinic 9	873	0.17
Tucker (2020)	Zambia	Clinic 10	477	0.16
*Within Study Average*				**0.70**
*Cross-Study Equipment and Capital Average*				**1.68**
**Laboratory**				

McBain (2017)	Malawi	Neno District	6,541	**1.08**
Chou (2007)	Uganda	Kampala RCT Study	1,420	**75.71**
Cianci (2014)	Burkina Faso	Yerelon Clinic	187	**185.56**
Tucker (2020)	Zambia	Clinic 1	9,104	33.88
Tucker (2020)	Zambia	Clinic 2	8,050	12.98
Tucker (2020)	Zambia	Clinic 3	6,410	18.08
Tucker (2020)	Zambia	Clinic 4	5,127	16.41
Tucker (2020)	Zambia	Clinic 5	4,597	11.26
Tucker (2020)	Zambia	Clinic 6	3,094	31.43
Tucker (2020)	Zambia	Clinic 7	3,080	9.80
Tucker (2020)	Zambia	Clinic 8	1,076	3.75
Tucker (2020)	Zambia	Clinic 9	873	5.53
Tucker (2020)	Zambia	Clinic 10	477	2.12
*Within Study Average*				**19.15**
*Cross-Study Laboratory Average*				**19.01**
**Supplies**				

Rout (2019)	India	Ahmednagar	58,393	1.06
Rout (2019)	India	Jalna	1,500	26.41
Rout (2019) ^†^	India	Bhandara	13,258	2.35
Rout (2019) ^†^	India	Kolhapur	32,122	1.18
Rout (2019) ^†^	India	Akola	3,774	6.94
Rout (2019) ^†^	India	Pandharpur	5,762	10.80
*Within Study Average*				**2.26**
McBain (2017)	Malawi	Neno District	6,541	**16.86**
Tucker (2020)	Zambia	Clinic 1	9,104	19.07
Tucker (2020)	Zambia	Clinic 2	8,050	0.59
Tucker (2020)	Zambia	Clinic 3	6,410	8.07
Tucker (2020)	Zambia	Clinic 4	5,127	1.85
Tucker (2020)	Zambia	Clinic 5	4,597	1.44
Tucker (2020)	Zambia	Clinic 6	3,094	4.17
Tucker (2020)	Zambia	Clinic 7	3,080	1.24
Tucker (2020)	Zambia	Clinic 8	1,076	9.84
Tucker (2020)	Zambia	Clinic 9	873	10.96
Tucker (2020)	Zambia	Clinic 10	477	4.88
*Within Study Average*				**6.81**
*Cross-Study Supplies Average*				**4.01**
**ART & Medicines**				

Rout (2019)	India	Ahmednagar	58,393	78.80
Rout (2019)	India	Jalna	1,500	249.21
Rout (2019) ^†^	India	Bhandara	13,258	172.88
Rout (2019) ^†^	India	Kolhapur	32,122	89.02
Rout (2019) ^†^	India	Akola	3,774	538.99
Rout (2019) ^†^	India	Pandharpur	5,762	547.63
*Within Study Average*				**133.41**
Chou (2007)	Uganda	Kampala RCT Study	1,420	**117.97**
McBain (2017)	Malawi	Neno District	6,541	**140.32**
Cianci (2014)	Burkina Faso	Yerelon Clinic	187	**378.82**
Tucker (2020)	Zambia	Clinic 1	9,104	97.13
Tucker (2020)	Zambia	Clinic 2	8,050	143.26
Tucker (2020)	Zambia	Clinic 3	6,410	103.99
Tucker (2020)	Zambia	Clinic 4	5,127	62.38
Tucker (2020)	Zambia	Clinic 5	4,597	71.92
Tucker (2020)	Zambia	Clinic 6	3,094	90.58
Tucker (2020)	Zambia	Clinic 7	3,080	109.92
Tucker (2020)	Zambia	Clinic 8	1,076	108.88
Tucker (2020)	Zambia	Clinic 9	873	45.06
Tucker (2020)	Zambia	Clinic 10	477	66.87
*Within Study Average*				**99.35**
Chou (2007)	Uganda	Kampala RCT Study	1,420	**14.63**
McBain (2017)	Malawi	Neno District	6,541	**1.89**
Cianci (2014)	Burkina Faso	Yerelon Clinic	187	**17.97**
*Cross-Study ART & Medicines Average*				**125.41**

***Cross-Study Total Cost***				**176.71**

* simple average

† author calculation for sample size.

ART denotes antiretroviral therapy.

**Table 4.** Annual Per Patient Costs for Nutrition Studies by Input Cost Categories (In US$, 2020)

First Author (Year)	Country	Type of Cost Unit (Health Center, Department)	Sample Size	Mean Unit Cost Per Capita

**Human Resources**				

Puett (2013)	Bangladesh	Community Treatment	724	49.16
Puett (2013)	Bangladesh	Inpatient Treatment	633	26.85
*Within Study Average*				**51.80**
Waters (2006)	Peru	Intervention Area	187	**20.47**
Puett (2013)	Chad	Food Assistance	1,071	**160.97**
Levin (2019)	Kenya	NGO 1	3,281	4.00
Levin (2019)	Kenya	NGO 2	3,281	5.73
Within Study Average*				**4.86**
Rogers (2019)	Pakistan	Intervention	425	141.85
Rogers (2019)	Pakistan	Control	393	140.21
*Within Study Average*				**141.06**
Puett (2014)	Zimbabwe	LIG	171	2,166.71
Puett (2014)	Zimbabwe	Comparator Households	45	473.02
*Within Study Average*				**1,902.33**
Rogers (2018)	Mali	Intervention	617	160.46
Rogers (2018)	Mali	Control	212	300.79
*Within Study Average*				**196.34**
*Cross-Study Human Resources Average*				**120.17**
**Equipment and Capital**				

Puett (2013)	Bangladesh	Community Treatment	724	1.27
Puett (2013)	Bangladesh	Inpatient Treatment	633	1.46
*Within Study Average*				**1.36**
Levin (2019)	Kenya	NGO 1	3,281	1.36
Levin (2019)	Kenya	NGO 2	3,281	0.72
Within Study Average*				**1.04**
*Cross-Study Equipment and Capital Average*				**1.13**
**Training**				

Waters (2006)	Peru	Intervention Area	187	**23.03**
Puett (2014)	Zimbabwe	LIG	171	1,152.26
Puett (2014)	Zimbabwe	Comparator Households	45	424.66
*Within Study Average*				**1,000.67**
Rogers (2018)	Mali	Intervention	617	16.88
Rogers (2018)	Mali	Control	212	39.34
*Within Study Average*				**22.62**
*Cross-Study Training Average*				**194.16**
**Logistics**				

Puett (2013)	Bangladesh	Community Treatment	724	34.32
Puett (2013)	Bangladesh	Inpatient Treatment	633	16.23
*Within Study Average*				**25.88**
Waters (2006)	Peru	Intervention Area	187	**4.75**
Rogers (2019)	Pakistan	Intervention	425	35.46
Rogers (2019)	Pakistan	Control	393	9.37
*Within Study Average*				**22.92**
Puett (2013)	Chad	Food Assistance	1,071	**16.32**
Rogers (2018)	Mali	Intervention	617	18.95
Rogers (2018)	Mali	Control	212	44.39
*Within Study Average*				**25.46**
*Cross-Study Logistics Average*				**27.19**
**Service Delivery**				

Rogers (2019)	Pakistan	Intervention	425	47.80
Rogers (2019)	Pakistan	Control	393	62.09
*Within Study Average*				**54.67**
Waters (2006)	Peru	Intervention Area	187	261.85
Waters (2006)	Peru	Control Area	187	304.16
Within Study Average*				**283.00**
Puett (2013)	Chad	Food Assistance	1,071	**43.74**
*Cross-Study Service Delivery Average*				**69.60**
**Supplies**				

Puett (2013)	Bangladesh	Community Treatment	724	**49.49**
Rogers (2019)	Pakistan	Intervention	425	51.42
Rogers (2019)	Pakistan	Intervention	393	51.60
*Within Study Average*				**51.51**
Levin (2019)	Kenya	NGO 1	3,281	2.53
Levin (2019)	Kenya	NGO 2	3,281	3.75
Within Study Average*				**3.14**
Puett (2013)	Chad	Food Assistance	1,071	**830.88**
Rogers (2018)	Mali	Intervention	617	32.40
Rogers (2018)	Mali	Control	212	30.00
*Within Study Average*				**31.84**
*Cross-Study Supplies Average*				**149.42**

***Cross-Study Total Cost***				**561.68**

* simple average

**Table 5.** Annual Per Patient Costs for Primary Care Studies by Input Cost Categories (In US$, 2020)

First Author (Year)	Country	Type of Cost Unit (Health Center, Department)	Sample Size	Mean Unit Cost Per Capita

**Human Resources**				

Deo (2019)	India	Patna	8,648	26.11
Deo (2019)	India	Mumbai	6,881	70.21
Deo (2019)	India	Mehsana	1,414	0.30
*Within Study Average*				**41.87**
Beauge (2018)	Burkina Faso	Design Phase	102,609	0.07
Beauge (2018)	Burkina Faso	Implementation Phase	102,609	3.12
Within Study Average*				**1.60**
Prinja (2016)	India	Primary Health Centers	37,635	153.14
Prinja (2016)	India	Community Health Centers	147,941	130.28
*Within Study Average*				**134.92**
Hussain (2006)	Pakistan	Gov’t PHC; PNA^+^	316	0.16
Hussain (2006)	Pakistan	Gov’t PHC; Severe PNA^+^	20	0.17
Hussain (2006)	Pakistan	AKHSP PHC; PNA^+^	157	0.13
Hussain (2006)	Pakistan	AKHSP PHC; Severe PNA^+^	3	0.13
*Within Study Average*				**0.15**
*Cross-Study Human Resources Average*				**84.78**
**Equipment and Capital**				

Deo (2019)	India	Patna	8,648	**0.29**
Prinja (2016)	India	Primary Health Centers	37,635	27.73
Prinja (2016)	India	Community Health Centers	147,941	17.93
*Within Study Average*				**19.92**
Hussain (2006)	Pakistan	Gov’t PHC; PNA^+^	316	0.10
Hussain (2006)	Pakistan	Gov’t PHC; Severe PNA^+^	20	0.10
Hussain (2006)	Pakistan	AKHSP PHC; PNA^+^	157	0.18
Hussain (2006)	Pakistan	AKHSP PHC; Severe PNA^+^	3	0.19
*Within Study Average*				**0.13**
*Cross-Study Equipment and Capital Average*				**19.87**
**Supplies**				

Deo (2019)	India	Mumbai	6,881	11.35
Deo (2019)	India	Mehsana	1,414	11.85
*Within Study Average*				**11.44**
Beauge (2018)	Burkina Faso	Design Phase	102,609	0.01
Beauge (2018)	Burkina Faso	Implementation Phase	102,609	0.89
Within Study Average*				**0.45**
Prinja (2016)	India	Primary Health Centers	37,635	14.10
Prinja (2016)	India	Community Health Centers	147,941	11.43
*Within Study Average*				**11.97**
Hussain (2006)	Pakistan	Gov’t PHC; PNA^+^	316	0.02
Hussain (2006)	Pakistan	Gov’t PHC; Severe PNA^+^	20	0.02
Hussain (2006)	Pakistan	AKHSP PHC; PNA^+^	157	0.01
Hussain (2006)	Pakistan	AKHSP PHC; Severe PNA^+^	3	0.01
*Within Study Average*				**0.02**
*Cross-Study Supplies Average*				**6.59**
**Consumables/Medicines**				

Deo (2019)	India	Patna	8,648	0.01
Deo (2019)	India	Mumbai	6,881	0.01
Deo (2019)	India	Mehsana	1,414	0.06
*Within Study Average*				**0.02**
Beauge (2018)	Burkina Faso	Design Phase	102,609	0.003
Beauge (2018)	Burkina Faso	Implementation Phase	102,609	2.06
Within Study Average*				**1.032**
Prinja (2016)	India	Primary Health Centers	37,635	55.02
Prinja (2016)	India	Community Health Centers	147,941	20.50
*Within Study Average*				**27.50**
Hussain (2006)	Pakistan	Gov’t PHC; PNA^+^	316	0.07
Hussain (2006)	Pakistan	Gov’t PHC; Severe PNA^+^	20	0.07
*Within Study Average*				**0.07**
*Cross-Study Consumables/Medicines Average*				**17.05**
**Laboratory**				

Deo (2019)	India	Patna	8,648	0.04
Deo (2019)	India	Mumbai	6,881	0.04
*Within Study Average*				**0.04**
Prinja (2016)	India	Primary Health Centers	37,635	7.90
Prinja (2016)	India	Community Health Centers	147,941	6.69
*Within Study Average*				**6.94**
*Cross-Study Laboratory Average*				**6.41**
**Miscellaneous**				

Deo (2019)	India	Patna	8,648	5.37
Deo (2019)	India	Mumbai	6,881	5.80
Deo (2019)	India	Mehsana	1,414	19.71
*Within Study Average*				**6.74**
Prinja (2016)	India	Primary Health Centers	37,635	1.1
Prinja (2016)	India	Community Health Centers	147,941	0.28
*Within Study Average*				**0.45**
Hussain (2006)	Pakistan	Gov’t PHC; PNA^+^	316	0.004
Hussain (2006)	Pakistan	Gov’t PHC; Severe PNA^+^	20	0.004
Hussain (2006)	Pakistan	AKHSP PHC; PNA^+^	157	0.004
Hussain (2006)	Pakistan	AKHSP PHC; Severe PNA^+^	3	0.006
*Within Study Average*				**0.004**
*Cross-Study Supplies Average*				**0.97**

***Cross-Study Primary Care Total Cost***				**135.67**

* simple average

† author calculation for sample size

+ outpatient

PNA: pneumonia

**Table 6.** Annual Per Patient Overhead Costs for HIV/Nutrition/Primary Care Studies (In US$, 2020)

First Author (Year)	Country	Type of Cost Unit (Health Center, Department)	Sample Size	Mean Unit Cost Per Capita

**HIV Studies**				

Tucker (2020)	Zambia	Clinic 1	9,104	0.20
Tucker (2020)	Zambia	Clinic 2	8,050	0.92
Tucker (2020)	Zambia	Clinic 3	6,410	0.60
Tucker (2020)	Zambia	Clinic 4	5,127	0.36
Tucker (2020)	Zambia	Clinic 5	4,597	0.52
Tucker (2020)	Zambia	Clinic 6	3,094	0.41
Tucker (2020)	Zambia	Clinic 7	3,080	2.38
Tucker (2020)	Zambia	Clinic 8	1,076	1.95
Tucker (2020)	Zambia	Clinic 9	873	1.33
Tucker (2020)	Zambia	Clinic 10	477	0.78
*Within Study Average*				**12.10**
Rout (2019)	India	Ahmednagar	58,393	31.01
Rout (2019)	India	Jalna	1,500	268.09
Rout (2019) ^†^	India	Bhandara	13,258	28.22
Rout (2019) ^†^	India	Kolhapur	32,122	18.28
Rout (2019) ^†^	India	Akola	3,774	96.89
Rout (2019) ^†^	India	Pandharpur	5,762	298.40
*Within Study Average*				**45.81**
Cianci (2014)	Burkina Faso	Yerelon Clinic	187	**370.13**
*Cross-Study HIV Overhead Cost Average*				**37.20**
**Nutrition Studies**				

Puett (2013)	Bangladesh	Community Treatment	724	22.40
Puett (2013)	Bangladesh	Inpatient Treatment	633	14.88
*Within Study Average*				**18.89**
Rogers (2019)	Pakistan	Intervention	425	21.21
Rogers (2019)	Pakistan	Control	393	39.56
*Within Study Average*				**30.03**
Levin (2019)	Kenya	NGO 1	3,281	0.48
Levin (2019)	Kenya	NGO 2	3,281	3.40
Within Study Average*				**1.94**
Rogers (2018)	Mali	Intervention	617	16.29
Rogers (2018)	Mali	Control	212	14.70
*Within Study Average*				**15.88**
*Cross-Study Nutrition Overhead Cost Average*				**9.00**
**Primary Care Studies**				

Deo (2019)	India	Mumbai	6881	11.35
Deo (2019)	India	Mehsana	1414	11.85
*Within Study Average*				**11.44**
Beauge (2018)	Burkina Faso	Design Phase	102609	0.01
Beauge (2018)	Burkina Faso	Implementation Phase	102609	0.89
Within Study Average*				**0.45**
Prinja (2016)	India	Primary Health Centers	37635	14.10
Prinja (2016)	India	Community Health Centers	147941	11.43
*Within Study Average*				**11.97**
Hussain (2006)	Pakistan	Gov’t PHC; PNA^+^	316	0.02
Hussain (2006)	Pakistan	Gov’t PHC; Severe PNA^+^	20	0.02
Hussain (2006)	Pakistan	AKHSP PHC; PNA^+^	157	0.01
Hussain (2006)	Pakistan	AKHSP PHC; Severe PNA^+^	3	0.01
*Within Study Average*				**0.02**
*Cross-Study Primary Care Overhead Cost Average*				**7.96**

* simple average

† author calculation for sample size

+ outpatient

PNA: pneumonia
