# Supplementary material for: Activity-based costing for HIV, primary care and nutrition services in low- and middle-income countries: A systematic literature review and synthesis
Source: J Glob Health Econ Policy. Author manuscript; Available in PMC 2022 Aug 16. (PMC9380588; doi:10.52872/001c.29068)
Supplement: Supplementary files [file NIHMS1751266-supplement-Supplementary_files.zip › all_files/table-1-search-strategies-for-key-databases.html]

| PubMed | Scopus | EconLit |
| --- | --- | --- |
| ("TD-ABC" OR "TDABC" OR “activity-based cost\*” OR “bottom-up cost\*” OR “unit costing” OR "patient-level cost\*") AND (("Economics"[MeSH] OR “economics”[subheading] OR Economic\* OR Cost\* OR Expenditure\* OR Accounting) OR (“Process map” OR “Process Assessment, Health Care”[MeSH]) OR ("Equipment and supplies"[MeSH] OR "Health Services Administration"[MeSH] OR "organization and administration"[subheading] OR "Task Performance and Analysis"[MeSH] OR "Workflow"[MeSH] OR "Job Satisfaction"[MeSH] OR Manag\* OR Staffing OR Equipment OR Supplies OR Personnel OR schedul\* OR "wait time\*" OR "prescrib\* pattern\*" OR satisfaction)) AND ( "2000/01/01"[PDat] : "2020/03/20"[PDat] ) | TITLE-ABS-KEY ( ( "TD-ABC" OR "activity-based cost\*" OR "bottom-up cost\*" OR "unit costing" OR "patient-level cost\*" ) AND ( health\* OR medic\* ) AND ( economic\* OR cost\* OR expenditure\* OR accounting OR "Process map" OR manag\* OR staffing OR equipment OR supplies OR personnel OR schedul\* OR "wait time\*" OR "prescrib\* pattern\*" OR satisfaction ) ) AND ( PUBYEAR > 1999 ) AND ( LANGUAGE ( English OR French OR Spanish ) ) | "TD-ABC" OR "TDABC" OR “activity-based cost\*” OR “bottom-up cost\*” OR “unit costing” OR "patient-level cost\*" |
