# Supplementary material for: Activity-based costing for HIV, primary care and nutrition services in low- and middle-income countries: A systematic literature review and synthesis
Source: J Glob Health Econ Policy. Author manuscript; Available in PMC 2022 Aug 16. (PMC9380588; doi:10.52872/001c.29068)
Supplement: Supplementary files [file NIHMS1751266-supplement-Supplementary_files.zip › all_files/table-2-summary-of-studies.html]

| **First Author** | **Year** | **Country** | **Income Level** | **Costing Method** | **Health Focus** | **Study Period** |
| --- | --- | --- | --- | --- | --- | --- |
| Chou | 2007 | Uganda | Low-Income | ABC | HIV | 2005-2006 |
| Cianci | 2014 | Burkina Faso | Low-Income | Bottom Up | HIV | 2010 |
| Levin | 2019 | Kenya | Lower-Middle Income | ABC | Nutrition | 2011-2013 |
| McBain | 2017 | Malawi | Low-Income | ABC/CEA | HIV | 2013-2014 |
| Puett | 2013 | Chad | Low-Income | ABC/CEA | Nutrition | 2010 |
| Puett | 2013 | Bangladesh | Lower-Middle Income | ABC/CEA | Nutrition | 2010 |
| Puett | 2014 | Zimbabwe | Lower-Middle Income | ABC/CEA | Nutrition | 2008-2011 |
| Rogers | 2019 | Pakistan | Lower-Middle Income | ABC/CEA | Nutrition | 2015-2016 |
| Rogers | 2018 | Mali | Low-Income | ABC/CEA | Nutrition | 2015-2016 |
| Rout | 2019 | India | Lower-Middle Income | Bottom Up | HIV | 2013-2015 |
| Tucker | 2020 | Zambia | Lower-Middle Income | ABC | HIV | 2015-2016 |
| Waters | 2006 | Peru | Lower-Middle Income | ABC | Nutrition | 2000-2001 |
| Deo | 2019 | India | Lower-Middle Income | ABC | Primary Care | 2014-2016 |
| Beauge | 2018 | Burkina Faso | Low-Income | ABC | Primary Care | 2014-2016 |
| Prinja | 2016 | India | Lower-Middle Income | Bottom Up | Primary Care | 2012-2013 |
| Hussain | 2006 | Pakistan | Lower-Middle Income | ABC | Primary Care | 2000-2001 |
