# Supplementary material for: Activity-based costing for HIV, primary care and nutrition services in low- and middle-income countries: A systematic literature review and synthesis
Source: J Glob Health Econ Policy. Author manuscript; Available in PMC 2022 Aug 16. (PMC9380588; doi:10.52872/001c.29068)
Supplement: Supplementary files [file NIHMS1751266-supplement-Supplementary_files.zip › all_files/table-4-annual-per-patient-costs-for-nutrition-studies-by-input-cost-categories-in-us-2020.html]

| **First Author (Year)** | **Country** | **Type of Cost Unit (Health Center, Department)** | **Sample Size** | **Mean Unit Cost Per Capita** |
| --- | --- | --- | --- | --- |
| **Human Resources** |  |  |  |  |
| Puett (2013) | Bangladesh | Community Treatment | 724 | 49.16 |
| Puett (2013) | Bangladesh | Inpatient Treatment | 633 | 26.85 |
| *Within Study Average* |  |  |  | **51.80** |
| Waters (2006) | Peru | Intervention Area | 187 | **20.47** |
| Puett (2013) | Chad | Food Assistance | 1,071 | **160.97** |
| Levin (2019) | Kenya | NGO 1 | 3,281 | 4.00 |
| Levin (2019) | Kenya | NGO 2 | 3,281 | 5.73 |
| *Within Study Average\** |  |  |  | **4.86** |
| Rogers (2019) | Pakistan | Intervention | 425 | 141.85 |
| Rogers (2019) | Pakistan | Control | 393 | 140.21 |
| *Within Study Average* |  |  |  | **141.06** |
| Puett (2014) | Zimbabwe | LIG | 171 | 2,166.71 |
| Puett (2014) | Zimbabwe | Comparator Households | 45 | 473.02 |
| *Within Study Average* |  |  |  | **1,902.33** |
| Rogers (2018) | Mali | Intervention | 617 | 160.46 |
| Rogers (2018) | Mali | Control | 212 | 300.79 |
| *Within Study Average* |  |  |  | **196.34** |
|  |  |  |  |  |
| *Cross-Study Human Resources Average* | | |  | **120.17** |
|  |  |  |  |  |
| **Equipment and Capital** | | | | |
| Puett (2013) | Bangladesh | Community Treatment | 724 | 1.27 |
| Puett (2013) | Bangladesh | Inpatient Treatment | 633 | 1.46 |
| *Within Study Average* |  |  |  | **1.36** |
| Levin (2019) | Kenya | NGO 1 | 3,281 | 1.36 |
| Levin (2019) | Kenya | NGO 2 | 3,281 | 0.72 |
| *Within Study Average\** |  |  |  | **1.04** |
|  |  |  |  |  |
| *Cross-Study Equipment and Capital Average* | | |  | **1.13** |
|  |  |  |  |  |
| **Training** |  |  |  |  |
| Waters (2006) | Peru | Intervention Area | 187 | **23.03** |
| Puett (2014) | Zimbabwe | LIG | 171 | 1,152.26 |
| Puett (2014) | Zimbabwe | Comparator Households | 45 | 424.66 |
| *Within Study Average* |  |  |  | **1,000.67** |
| Rogers (2018) | Mali | Intervention | 617 | 16.88 |
| Rogers (2018) | Mali | Control | 212 | 39.34 |
| *Within Study Average* |  |  |  | **22.62** |
|  |  |  |  |  |
| *Cross-Study Training Average* | | |  | **194.16** |
|  |  |  |  |  |
| **Logistics** |  |  |  |  |
| Puett (2013) | Bangladesh | Community Treatment | 724 | 34.32 |
| Puett (2013) | Bangladesh | Inpatient Treatment | 633 | 16.23 |
| *Within Study Average* |  |  |  | **25.88** |
| Waters (2006) | Peru | Intervention Area | 187 | **4.75** |
| Rogers (2019) | Pakistan | Intervention | 425 | 35.46 |
| Rogers (2019) | Pakistan | Control | 393 | 9.37 |
| *Within Study Average* |  |  |  | **22.92** |
| Puett (2013) | Chad | Food Assistance | 1,071 | **16.32** |
| Rogers (2018) | Mali | Intervention | 617 | 18.95 |
| Rogers (2018) | Mali | Control | 212 | 44.39 |
| *Within Study Average* |  |  |  | **25.46** |
| *Cross-Study Logistics Average* | | |  | **27.19** |
| **Service Delivery** |  |  |  |  |
| Rogers (2019) | Pakistan | Intervention | 425 | 47.80 |
| Rogers (2019) | Pakistan | Control | 393 | 62.09 |
| *Within Study Average* |  |  |  | **54.67** |
| Waters (2006) | Peru | Intervention Area | 187 | 261.85 |
| Waters (2006) | Peru | Control Area | 187 | 304.16 |
| *Within Study Average\** |  |  |  | **283.00** |
| Puett (2013) | Chad | Food Assistance | 1,071 | **43.74** |
| *Cross-Study Service Delivery Average* | | |  | **69.60** |
| **Supplies** |  |  |  |  |
| Puett (2013) | Bangladesh | Community Treatment | 724 | **49.49** |
| Rogers (2019) | Pakistan | Intervention | 425 | 51.42 |
| Rogers (2019) | Pakistan | Intervention | 393 | 51.60 |
| *Within Study Average* |  |  |  | **51.51** |
| Levin (2019) | Kenya | NGO 1 | 3,281 | 2.53 |
| Levin (2019) | Kenya | NGO 2 | 3,281 | 3.75 |
| *Within Study Average\** |  |  |  | **3.14** |
| Puett (2013) | Chad | Food Assistance | 1,071 | **830.88** |
| Rogers (2018) | Mali | Intervention | 617 | 32.40 |
| Rogers (2018) | Mali | Control | 212 | 30.00 |
| *Within Study Average* |  |  |  | **31.84** |
|  |  |  |  |  |
| *Cross-Study Supplies Average* | | |  | **149.42** |
|  |  |  |  |  |
| ***Cross-Study Total Cost*** | | |  | **561.68** |
