# Supplementary material for: Activity-based costing for HIV, primary care and nutrition services in low- and middle-income countries: A systematic literature review and synthesis
Source: J Glob Health Econ Policy. Author manuscript; Available in PMC 2022 Aug 16. (PMC9380588; doi:10.52872/001c.29068)
Supplement: Supplementary files [file NIHMS1751266-supplement-Supplementary_files.zip › all_files/table-5-annual-per-patient-costs-for-primary-care-studies-by-input-cost-categories-in-us-2020.html]

| **First Author (Year)** | **Country** | **Type of Cost Unit (Health Center, Department)** | **Sample Size** | **Mean Unit Cost Per Capita** |
| --- | --- | --- | --- | --- |
| **Human Resources** |  |  |  |  |
| Deo (2019) | India | Patna | 8,648 | 26.11 |
| Deo (2019) | India | Mumbai | 6,881 | 70.21 |
| Deo (2019) | India | Mehsana | 1,414 | 0.30 |
| *Within Study Average* |  |  |  | **41.87** |
| Beauge (2018) | Burkina Faso | Design Phase | 102,609 | 0.07 |
| Beauge (2018) | Burkina Faso | Implementation Phase | 102,609 | 3.12 |
| *Within Study Average\** |  |  |  | **1.60** |
| Prinja (2016) | India | Primary Health Centers | 37,635 | 153.14 |
| Prinja (2016) | India | Community Health Centers | 147,941 | 130.28 |
| *Within Study Average* |  |  |  | **134.92** |
| Hussain (2006) | Pakistan | Gov’t PHC; PNA⁺ | 316 | 0.16 |
| Hussain (2006) | Pakistan | Gov’t PHC; Severe PNA⁺ | 20 | 0.17 |
| Hussain (2006) | Pakistan | AKHSP PHC; PNA⁺ | 157 | 0.13 |
| Hussain (2006) | Pakistan | AKHSP PHC; Severe PNA⁺ | 3 | 0.13 |
| *Within Study Average* |  |  |  | **0.15** |
| *Cross-Study Human Resources Average* | | |  | **84.78** |
| **Equipment and Capital** |  |  |  |  |
| Deo (2019) | India | Patna | 8,648 | **0.29** |
| Prinja (2016) | India | Primary Health Centers | 37,635 | 27.73 |
| Prinja (2016) | India | Community Health Centers | 147,941 | 17.93 |
| *Within Study Average* |  |  |  | **19.92** |
| Hussain (2006) | Pakistan | Gov’t PHC; PNA⁺ | 316 | 0.10 |
| Hussain (2006) | Pakistan | Gov’t PHC; Severe PNA⁺ | 20 | 0.10 |
| Hussain (2006) | Pakistan | AKHSP PHC; PNA⁺ | 157 | 0.18 |
| Hussain (2006) | Pakistan | AKHSP PHC; Severe PNA⁺ | 3 | 0.19 |
| *Within Study Average* |  |  |  | **0.13** |
| *Cross-Study Equipment and Capital Average* | | |  | **19.87** |
| **Supplies** |  |  |  |  |
| Deo (2019) | India | Mumbai | 6,881 | 11.35 |
| Deo (2019) | India | Mehsana | 1,414 | 11.85 |
| *Within Study Average* |  |  |  | **11.44** |
| Beauge (2018) | Burkina Faso | Design Phase | 102,609 | 0.01 |
| Beauge (2018) | Burkina Faso | Implementation Phase | 102,609 | 0.89 |
| *Within Study Average\** |  |  |  | **0.45** |
| Prinja (2016) | India | Primary Health Centers | 37,635 | 14.10 |
| Prinja (2016) | India | Community Health Centers | 147,941 | 11.43 |
| *Within Study Average* |  |  |  | **11.97** |
| Hussain (2006) | Pakistan | Gov’t PHC; PNA⁺ | 316 | 0.02 |
| Hussain (2006) | Pakistan | Gov’t PHC; Severe PNA⁺ | 20 | 0.02 |
| Hussain (2006) | Pakistan | AKHSP PHC; PNA⁺ | 157 | 0.01 |
| Hussain (2006) | Pakistan | AKHSP PHC; Severe PNA⁺ | 3 | 0.01 |
| *Within Study Average* |  |  |  | **0.02** |
| *Cross-Study Supplies Average* | | |  | **6.59** |
| **Consumables/Medicines** |  |  |  |  |
| Deo (2019) | India | Patna | 8,648 | 0.01 |
| Deo (2019) | India | Mumbai | 6,881 | 0.01 |
| Deo (2019) | India | Mehsana | 1,414 | 0.06 |
| *Within Study Average* |  |  |  | **0.02** |
| Beauge (2018) | Burkina Faso | Design Phase | 102,609 | 0.003 |
| Beauge (2018) | Burkina Faso | Implementation Phase | 102,609 | 2.06 |
| *Within Study Average\** |  |  |  | **1.032** |
| Prinja (2016) | India | Primary Health Centers | 37,635 | 55.02 |
| Prinja (2016) | India | Community Health Centers | 147,941 | 20.50 |
| *Within Study Average* |  |  |  | **27.50** |
| Hussain (2006) | Pakistan | Gov’t PHC; PNA⁺ | 316 | 0.07 |
| Hussain (2006) | Pakistan | Gov’t PHC; Severe PNA⁺ | 20 | 0.07 |
| *Within Study Average* |  |  |  | **0.07** |
| *Cross-Study Consumables/Medicines Average* | | |  | **17.05** |
| **Laboratory** |  |  |  |  |
| Deo (2019) | India | Patna | 8,648 | 0.04 |
| Deo (2019) | India | Mumbai | 6,881 | 0.04 |
| *Within Study Average* | | | | **0.04** |
| Prinja (2016) | India | Primary Health Centers | 37,635 | 7.90 |
| Prinja (2016) | India | Community Health Centers | 147,941 | 6.69 |
| *Within Study Average* |  |  |  | **6.94** |
| *Cross-Study Laboratory Average* | | |  | **6.41** |
| **Miscellaneous** |  |  |  |  |
| Deo (2019) | India | Patna | 8,648 | 5.37 |
| Deo (2019) | India | Mumbai | 6,881 | 5.80 |
| Deo (2019) | India | Mehsana | 1,414 | 19.71 |
| *Within Study Average* |  |  |  | **6.74** |
| Prinja (2016) | India | Primary Health Centers | 37,635 | 1.1 |
| Prinja (2016) | India | Community Health Centers | 147,941 | 0.28 |
| *Within Study Average* |  |  |  | **0.45** |
| Hussain (2006) | Pakistan | Gov’t PHC; PNA⁺ | 316 | 0.004 |
| Hussain (2006) | Pakistan | Gov’t PHC; Severe PNA⁺ | 20 | 0.004 |
| Hussain (2006) | Pakistan | AKHSP PHC; PNA⁺ | 157 | 0.004 |
| Hussain (2006) | Pakistan | AKHSP PHC; Severe PNA⁺ | 3 | 0.006 |
| *Within Study Average* |  |  |  | **0.004** |
| *Cross-Study Supplies Average* | | |  | **0.97** |
| ***Cross-Study Primary Care Total Cost*** | | |  | **135.67** |
