# Supplementary material for: Activity-based costing for HIV, primary care and nutrition services in low- and middle-income countries: A systematic literature review and synthesis
Source: J Glob Health Econ Policy. Author manuscript; Available in PMC 2022 Aug 16. (PMC9380588; doi:10.52872/001c.29068)
Supplement: Supplementary files [file NIHMS1751266-supplement-Supplementary_files.zip › all_files/table-3-annual-per-patient-costs-for-hiv-studies-by-input-cost-categories-in-us-2020.html]

| **First Author (Year)** | **Country** | **Type of Cost Unit (Health Center, Department)** | **Sample Size** | **Mean Per Patient Unit Cost** |
| --- | --- | --- | --- | --- |
| **Human Resources** |  |  |  |  |
| Rout (2019) | India | Ahmednagar | 58,393 | 7.62 |
| Rout (2019) | India | Jalna | 1,500 | 137.23 |
| **Rout (2019)†** | India | Bhandara | 13,258 | 28.21 |
| **Rout (2019)†** | India | Kolhapur | 32,122 | 13.87 |
| **Rout (2019)†** | India | Akola | 3,774 | 86.24 |
| **Rout (2019)†** | India | Pandharpur | 5,762 | 61.21 |
| *Within Study Average* |  |  |  | **18.71** |
| Chou (2007) | Uganda | Kampala RCT Study | 1,420 | **782.51** |
| McBain (2017) | Malawi | Neno District | 6,541 | **140.10** |
| Cianci (2014) | Burkina Faso | Direct/Yerelon Clinic | 187 | 69.02 |
| Cianci (2014) | Burkina Faso | Non-Direct/Yerelon Clinic | 187 | 68.27 |
| *Within Study Average\** |  |  |  | **68.65** |
| Tucker (2020) | Zambia | Clinic 1 | 9,104 | 4.33 |
| Tucker (2020) | Zambia | Clinic 2 | 8,050 | 5.44 |
| Tucker (2020) | Zambia | Clinic 3 | 6,410 | 4.74 |
| Tucker (2020) | Zambia | Clinic 4 | 5,127 | 3.15 |
| Tucker (2020) | Zambia | Clinic 5 | 4,597 | 5.57 |
| Tucker (2020) | Zambia | Clinic 6 | 3,094 | 4.30 |
| Tucker (2020) | Zambia | Clinic 7 | 3,080 | 4.21 |
| Tucker (2020) | Zambia | Clinic 8 | 1,076 | 9.23 |
| Tucker (2020) | Zambia | Clinic 9 | 873 | 3.11 |
| Tucker (2020) | Zambia | Clinic 10 | 477 | 3.11 |
| *Within Study Average* |  |  |  | **4.67** |
| *Cross-Study Human Resources Average* | | | | **26.60** |
| **Equipment and Capital** |  |  |  |  |
| Rout (2019) | India | Ahmednagar | 58,393 | 0.47 |
| Rout (2019) | India | Jalna | 1,500 | 25.08 |
| **Rout (2019)†** | India | Bhandara | 13,258 | 2.40 |
| **Rout (2019)†** | India | Kolhapur | 32,122 | 0.56 |
| **Rout (2019)†** | India | Akola | 3,774 | 9.23 |
| **Rout (2019)†** | India | Pandharpur | 5,762 | 4.47 |
| *Within Study Average* |  |  |  | **1.53** |
| Chou (2007) | Uganda | Kampala RCT Study | 1,420 | **15.40** |
| McBain (2017) | Malawi | Neno District | 6,541 | **7.60** |
| Tucker (2020) | Zambia | Clinic 1 | 9,104 | 0.61 |
| Tucker (2020) | Zambia | Clinic 2 | 8,050 | 0.57 |
| Tucker (2020) | Zambia | Clinic 3 | 6,410 | 0.32 |
| Tucker (2020) | Zambia | Clinic 4 | 5,127 | 0.29 |
| Tucker (2020) | Zambia | Clinic 5 | 4,597 | 0.39 |
| Tucker (2020) | Zambia | Clinic 6 | 3,094 | 0.42 |
| Tucker (2020) | Zambia | Clinic 7 | 3,080 | 2.67 |
| Tucker (2020) | Zambia | Clinic 8 | 1,076 | 3.67 |
| Tucker (2020) | Zambia | Clinic 9 | 873 | 0.17 |
| Tucker (2020) | Zambia | Clinic 10 | 477 | 0.16 |
| *Within Study Average* |  |  |  | **0.70** |
|  |  |  |  |  |
| *Cross-Study Equipment and Capital Average* | | |  | **1.68** |
|  |  |  |
| **Laboratory** |  |  |  |  |
| McBain (2017) | Malawi | Neno District | 6,541 | **1.08** |
| Chou (2007) | Uganda | Kampala RCT Study | 1,420 | **75.71** |
| Cianci (2014) | Burkina Faso | Yerelon Clinic | 187 | **185.56** |
| Tucker (2020) | Zambia | Clinic 1 | 9,104 | 33.88 |
| Tucker (2020) | Zambia | Clinic 2 | 8,050 | 12.98 |
| Tucker (2020) | Zambia | Clinic 3 | 6,410 | 18.08 |
| Tucker (2020) | Zambia | Clinic 4 | 5,127 | 16.41 |
| Tucker (2020) | Zambia | Clinic 5 | 4,597 | 11.26 |
| Tucker (2020) | Zambia | Clinic 6 | 3,094 | 31.43 |
| Tucker (2020) | Zambia | Clinic 7 | 3,080 | 9.80 |
| Tucker (2020) | Zambia | Clinic 8 | 1,076 | 3.75 |
| Tucker (2020) | Zambia | Clinic 9 | 873 | 5.53 |
| Tucker (2020) | Zambia | Clinic 10 | 477 | 2.12 |
| *Within Study Average* |  |  |  | **19.15** |
|  |  |  |  |  |
| *Cross-Study Laboratory Average* | | | | **19.01** |
|  |  |  |  |  |
| **Supplies** |  |  |  |  |
| Rout (2019) | India | Ahmednagar | 58,393 | 1.06 |
| Rout (2019) | India | Jalna | 1,500 | 26.41 |
| **Rout (2019)†** | India | Bhandara | 13,258 | 2.35 |
| **Rout (2019)†** | India | Kolhapur | 32,122 | 1.18 |
| **Rout (2019)†** | India | Akola | 3,774 | 6.94 |
| **Rout (2019)†** | India | Pandharpur | 5,762 | 10.80 |
| *Within Study Average* |  |  |  | **2.26** |
| McBain (2017) | Malawi | Neno District | 6,541 | **16.86** |
| Tucker (2020) | Zambia | Clinic 1 | 9,104 | 19.07 |
| Tucker (2020) | Zambia | Clinic 2 | 8,050 | 0.59 |
| Tucker (2020) | Zambia | Clinic 3 | 6,410 | 8.07 |
| Tucker (2020) | Zambia | Clinic 4 | 5,127 | 1.85 |
| Tucker (2020) | Zambia | Clinic 5 | 4,597 | 1.44 |
| Tucker (2020) | Zambia | Clinic 6 | 3,094 | 4.17 |
| Tucker (2020) | Zambia | Clinic 7 | 3,080 | 1.24 |
| Tucker (2020) | Zambia | Clinic 8 | 1,076 | 9.84 |
| Tucker (2020) | Zambia | Clinic 9 | 873 | 10.96 |
| Tucker (2020) | Zambia | Clinic 10 | 477 | 4.88 |
| *Within Study Average* |  |  |  | **6.81** |
|  |  |  |  |  |
| *Cross-Study Supplies Average* | | |  | **4.01** |
| **ART & Medicines** |  |  |  |  |
| Rout (2019) | India | Ahmednagar | 58,393 | 78.80 |
| Rout (2019) | India | Jalna | 1,500 | 249.21 |
| **Rout (2019)†** | India | Bhandara | 13,258 | 172.88 |
| **Rout (2019)†** | India | Kolhapur | 32,122 | 89.02 |
| **Rout (2019)†** | India | Akola | 3,774 | 538.99 |
| **Rout (2019)†** | India | Pandharpur | 5,762 | 547.63 |
| *Within Study Average* |  |  |  | **133.41** |
| Chou (2007) | Uganda | Kampala RCT Study | 1,420 | **117.97** |
| McBain (2017) | Malawi | Neno District | 6,541 | **140.32** |
| Cianci (2014) | Burkina Faso | Yerelon Clinic | 187 | **378.82** |
| Tucker (2020) | Zambia | Clinic 1 | 9,104 | 97.13 |
| Tucker (2020) | Zambia | Clinic 2 | 8,050 | 143.26 |
| Tucker (2020) | Zambia | Clinic 3 | 6,410 | 103.99 |
| Tucker (2020) | Zambia | Clinic 4 | 5,127 | 62.38 |
| Tucker (2020) | Zambia | Clinic 5 | 4,597 | 71.92 |
| Tucker (2020) | Zambia | Clinic 6 | 3,094 | 90.58 |
| Tucker (2020) | Zambia | Clinic 7 | 3,080 | 109.92 |
| Tucker (2020) | Zambia | Clinic 8 | 1,076 | 108.88 |
| Tucker (2020) | Zambia | Clinic 9 | 873 | 45.06 |
| Tucker (2020) | Zambia | Clinic 10 | 477 | 66.87 |
| *Within Study Average* |  |  |  | **99.35** |
| Chou (2007) | Uganda | Kampala RCT Study | 1,420 | **14.63** |
| McBain (2017) | Malawi | Neno District | 6,541 | **1.89** |
| Cianci (2014) | Burkina Faso | Yerelon Clinic | 187 | **17.97** |
|  |  |  |  |  |
| *Cross-Study ART & Medicines Average* | | |  | **125.41** |
|  |  |  |  |  |
| ***Cross-Study Total Cost*** | | | | **176.71** |
