# Supplementary material for: Activity-based costing for HIV, primary care and nutrition services in low- and middle-income countries: A systematic literature review and synthesis
Source: J Glob Health Econ Policy. Author manuscript; Available in PMC 2022 Aug 16. (PMC9380588; doi:10.52872/001c.29068)
Supplement: Supplementary files [file NIHMS1751266-supplement-Supplementary_files.zip › all_files/table-6-annual-per-patient-overhead-costs-for-hiv-nutrition-primary-care-studies-in-us-2020.html]

| **First Author (Year)** | **Country** | **Type of Cost Unit (Health Center, Department)** | **Sample Size** | **Mean Unit Cost Per Capita** |
| --- | --- | --- | --- | --- |
| **HIV Studies** |  |  |  |  |
| Tucker (2020) | Zambia | Clinic 1 | 9,104 | 0.20 |
| Tucker (2020) | Zambia | Clinic 2 | 8,050 | 0.92 |
| Tucker (2020) | Zambia | Clinic 3 | 6,410 | 0.60 |
| Tucker (2020) | Zambia | Clinic 4 | 5,127 | 0.36 |
| Tucker (2020) | Zambia | Clinic 5 | 4,597 | 0.52 |
| Tucker (2020) | Zambia | Clinic 6 | 3,094 | 0.41 |
| Tucker (2020) | Zambia | Clinic 7 | 3,080 | 2.38 |
| Tucker (2020) | Zambia | Clinic 8 | 1,076 | 1.95 |
| Tucker (2020) | Zambia | Clinic 9 | 873 | 1.33 |
| Tucker (2020) | Zambia | Clinic 10 | 477 | 0.78 |
| *Within Study Average* |  |  |  | **12.10** |
| Rout (2019) | India | Ahmednagar | 58,393 | 31.01 |
| Rout (2019) | India | Jalna | 1,500 | 268.09 |
| **Rout (2019)†** | India | Bhandara | 13,258 | 28.22 |
| **Rout (2019)†** | India | Kolhapur | 32,122 | 18.28 |
| **Rout (2019)†** | India | Akola | 3,774 | 96.89 |
| **Rout (2019)†** | India | Pandharpur | 5,762 | 298.40 |
| *Within Study Average* |  |  |  | **45.81** |
| Cianci (2014) | Burkina Faso | Yerelon Clinic | 187 | **370.13** |
| *Cross-Study HIV Overhead Cost Average* | | |  | **37.20** |
| **Nutrition Studies** |  |  |  |  |
| Puett (2013) | Bangladesh | Community Treatment | 724 | 22.40 |
| Puett (2013) | Bangladesh | Inpatient Treatment | 633 | 14.88 |
| *Within Study Average* |  |  |  | **18.89** |
| Rogers (2019) | Pakistan | Intervention | 425 | 21.21 |
| Rogers (2019) | Pakistan | Control | 393 | 39.56 |
| *Within Study Average* |  |  |  | **30.03** |
| Levin (2019) | Kenya | NGO 1 | 3,281 | 0.48 |
| Levin (2019) | Kenya | NGO 2 | 3,281 | 3.40 |
| *Within Study Average\** |  |  |  | **1.94** |
| Rogers (2018) | Mali | Intervention | 617 | 16.29 |
| Rogers (2018) | Mali | Control | 212 | 14.70 |
| *Within Study Average* |  |  |  | **15.88** |
| *Cross-Study Nutrition Overhead Cost Average* | | |  | **9.00** |
| **Primary Care Studies** |  |  |  |  |
| Deo (2019) | India | Mumbai | 6881 | 11.35 |
| Deo (2019) | India | Mehsana | 1414 | 11.85 |
| *Within Study Average* |  |  |  | **11.44** |
| Beauge (2018) | Burkina Faso | Design Phase | 102609 | 0.01 |
| Beauge (2018) | Burkina Faso | Implementation Phase | 102609 | 0.89 |
| *Within Study Average\** |  |  |  | **0.45** |
| Prinja (2016) | India | Primary Health Centers | 37635 | 14.10 |
| Prinja (2016) | India | Community Health Centers | 147941 | 11.43 |
| *Within Study Average* |  |  |  | **11.97** |
| Hussain (2006) | Pakistan | Gov’t PHC; PNA⁺ | 316 | 0.02 |
| Hussain (2006) | Pakistan | Gov’t PHC; Severe PNA⁺ | 20 | 0.02 |
| Hussain (2006) | Pakistan | AKHSP PHC; PNA⁺ | 157 | 0.01 |
| Hussain (2006) | Pakistan | AKHSP PHC; Severe PNA⁺ | 3 | 0.01 |
| *Within Study Average* |  |  |  | **0.02** |
| *Cross-Study Primary Care Overhead Cost Average* | | |  | **7.96** |
