# Supplementary figures and images for: Activity-based costing for HIV, primary care and nutrition services in low- and middle-income countries: A systematic literature review and synthesis
Source: J Glob Health Econ Policy. Author manuscript; Available in PMC 2022 Aug 16. (PMC9380588; doi:10.52872/001c.29068)

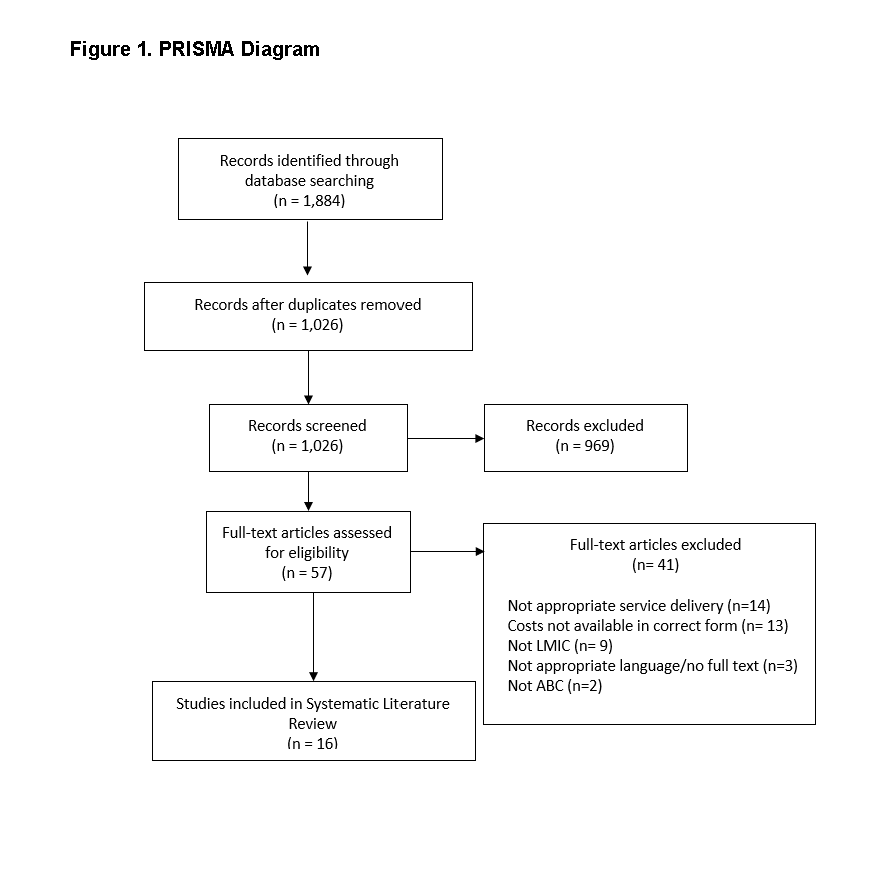

Supplement: Supplementary files [file NIHMS1751266-supplement-Supplementary_files.zip › all_files/abc_costing_figure_1.tiff]

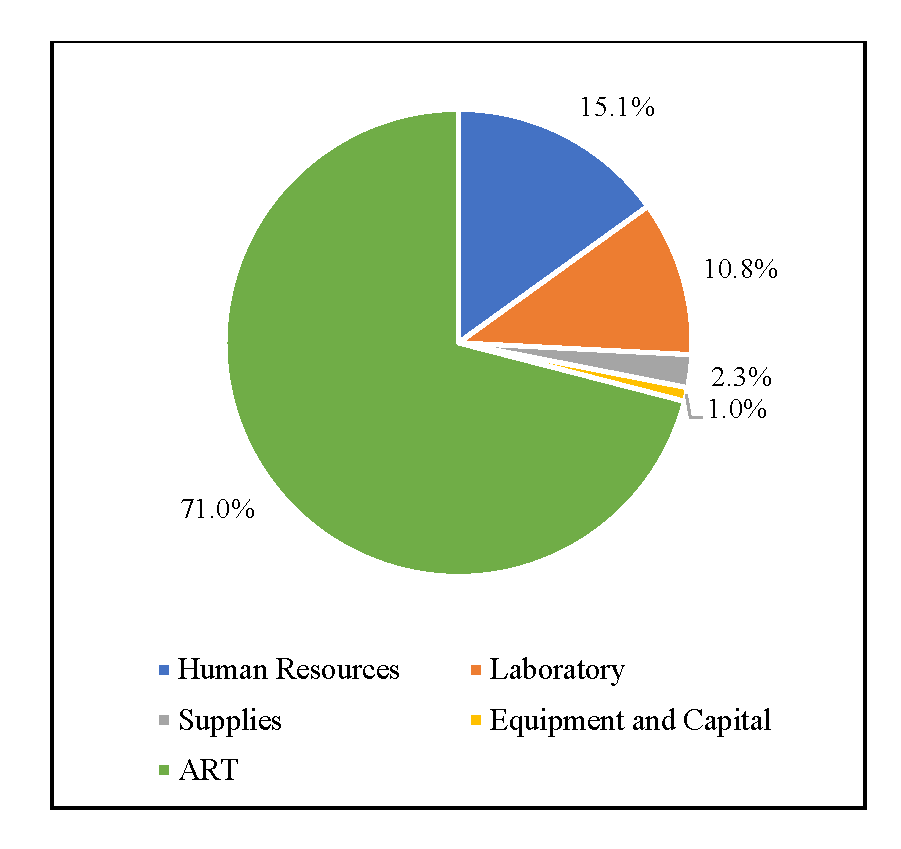

Supplement: Supplementary files [file NIHMS1751266-supplement-Supplementary_files.zip › all_files/abc_costing_figure_2.tiff]

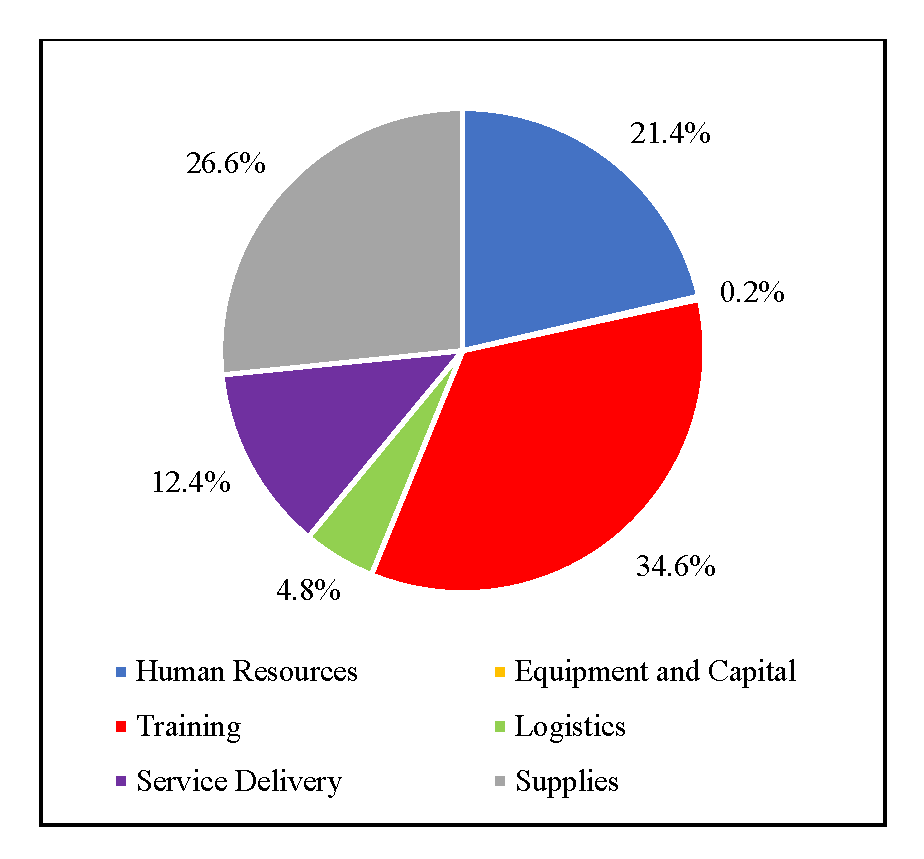

Supplement: Supplementary files [file NIHMS1751266-supplement-Supplementary_files.zip › all_files/abc_costing_figure_3.tiff]

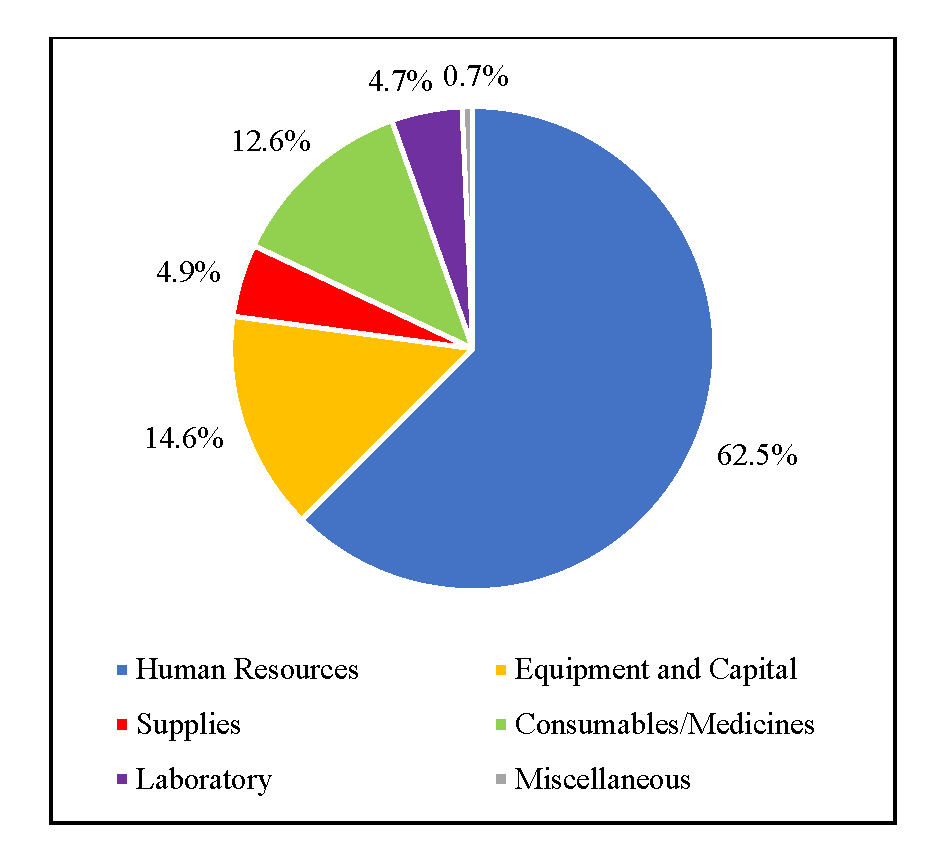

Supplement: Supplementary files [file NIHMS1751266-supplement-Supplementary_files.zip › all_files/abc_costing_figure_4.tiff]
